# Effectiveness of virtual reality-based nature interventions in promoting mental health outcomes among older adults with cognitive impairment: a systematic review and meta-regression analysis

**DOI:** 10.1080/00049530.2026.2679707

**Published:** 2026-06-15

**Authors:** Rashid Menhas, Hamzah M. Alghzawi, Muhammad Muddasar Saeed, Hasnain Ali

**Affiliations:** a School of Nursing, Shandong Xiehe University, Jinan, China; b Vanderbilt University Medical Center (VUMC), Nashville, TN, USA; c School of Nursing, Tennessee State University, Nashville, TN, USA; d Department of Laboratory Medicine, The First Affiliated Hospital of Henan University of Chinese Medicine, Zhengzhou, Henan, China; e Department of Public Health, Monroe University, New York, USA

**Keywords:** Virtual reality, nature exposure, cognitive impairment, older adults, mental health

## Abstract

**Objective:**

This systematic review and meta-regression evaluated virtual reality‑based nature interventions for older adults with cognitive impairment.

**Methods:**

Following PRISMA 2020 and a registered PROSPERO protocol (January 2000–December 2025), we identified randomised controlled trials from nine databases and two registries. Eligible interventions used simulated nature as the primary therapeutic component. Random‑effects models pooled standardised mean differences (SMDs) and risk ratios (RRs).

**Results:**

Twenty‑four RCTs (N = 1,534) were included. VR nature interventions significantly improved depression (SMD = −1.17), anxiety (−1.45), stress/distress (−1.38), apathy (−1.33), loneliness (−1.32), agitation (−1.69), and overall neuropsychiatric symptoms (−1.34). Quality of life improved (mental health‑related SMD = 1.26; global SMD = 1.14; mood/positive affect SMD = 1.18). Sleep quality improved (RR = 3.44). Engagement favoured VR (SMD = 1.07), while physiological stress markers showed mixed effects (SMD = −0.72). Dichotomous outcomes had no heterogeneity (I² = 0%); continuous outcomes showed substantial heterogeneity (I² > 90%). Meta‑regression found a modest positive association between intervention duration and effect size (β = 0.01/week).

**Conclusions:**

VR‑based nature interventions may improve mental health and quality of life in older adults with cognitive impairment. However, large effect sizes, high heterogeneity, and potential small‑study effects warrant cautious interpretation.

## KEY POINTS

What is already known about this topic:

(1) Neuropsychiatric symptoms, such as depression, anxiety, apathy, agitation, and loneliness, are highly prevalent in older adults with cognitive impairment and are associated with increased caregiver burden, functional decline, and limited response to pharmacological treatments.

(2) Exposure to natural environments has established mental health benefits; however, physical access is frequently restricted by mobility limitations, institutional settings, and safety concerns, prompting interest in simulated alternatives.

(3) Prior narrative reviews have documented variability in virtual reality – based nature interventions according to immersion level and content characteristics; however, no comprehensive quantitative synthesis has specifically focused on randomised evidence in older adults with cognitive impairment.

What this topic adds:

(1) This systematic review and meta-regression of 24 randomised controlled trials (*N* = 1,534) demonstrated large, consistent improvements across multiple domains, including depression (SMD −1.17), anxiety (SMD −1.45), agitation (SMD −1.69), loneliness (SMD −1.32), and mental health – related quality of life (SMD 1.26), with high engagement (SMD 1.07).

(2) Meta-regression identified intervention duration as a modest positive moderator (β≈+0.01 per week) of effect size, while revealing substantial heterogeneity (I2 > 90% for continuous outcomes) and small-study effects that warrant cautious interpretation of the results.

(3) The analysis provides the first pooled estimates of feasibility, safety, and evidence certainty (GRADE) for this population, offering actionable guidance for standardised content libraries, supervised delivery protocols, and future multicentre trials with longer follow-up.

## Introduction

Mental health symptoms among older adults with cognitive impairment represent a major public health and clinical care challenge (Fiske et al., 2009). Older adults experiencing mental health issues caused by cognitive impairment subsequently face major public health issues (Fiske et al., 2009; Kales et al., 2015). Current estimates indicate a large number of elderly individuals with mild cognitive impairment, with variations based on region and whether individuals qualify based on the diagnostic criteria (Salari et al., 2025). In addition, neuropsychiatric symptoms are present in every phase of dementia (depression, anxiety, and apathy) and demonstrate poor functional ability and higher complexity in clinical practice (Kales et al., 2015; Leung et al., 2021). Globally (within the United States), there has been a significant increase in the number of new cases of Alzheimer’s disease and related dementias. The major contributors are the ageing population and increased life expectancy, which also contribute to deaths and disabilities (2022; Hebert et al., 2013; Xiaopeng et al., 2025). Currently, empirical evidence supports the established relationship between emotional distress (affect) and future cognitive impairment outcomes, emphasising the need for scalable mental health treatments/programs that can be adapted for people with cognitive impairment (Fiske et al., 2009; Santabárbara et al., 2020). Currentlydementia care models focus on prevention and risk mitigation and include many different types of non-pharmacological treatments to assist with the reduction of symptoms and caregiver burden, regardless of the setting (Kales et al., 2015; Livingston et al., 2020; Oh et al., 2019).

The pharmacological management of neuropsychiatric symptoms in older, frail adults using medications has yielded minimal benefits, as many of these medications pose safety concerns (Kales et al., 2015; Livingston et al., 2020). Consequently, non-pharmacological interventions that can be delivered safely and consistently in care homes, clinics, and community settings are increasingly important. Thus, there is increasing interest in non-pharmacological interventions with viable delivery methods through long-term care and community services (Livingston et al., 2020; Lee, 2021; Prinz et al., 2024). Furthermore, spending time in natural settings has been shown to provide beneficial psychological results, and various studies have shown that when physical access to nature is not possible due to mobility issues, safety concerns, living in institutions, environmental barriers, weather conditions, and caregiver availability, simulated nature can also provide mental health benefits (Berman et al., 2008; Bratman et al., 2015; Browning et al., 2020; Prinz et al., 2024; Twohig-Bennett & Jones, 2018; Valtchanov et al., 2010; White et al., 2019; Yu et al., 2020). However, for many older adults with cognitive impairment, direct access to outdoor natural environments is limited by mobility impairment, safety concerns, institutional residence, environmental barriers, weather conditions, and caregiver availability (Huygelier et al., 2019). Therefore, virtual reality (VR) nature-based interventions are a promising alternative to provide individuals with a safe, standardised, and replicable experience of nature indoors through immersive (360°) and interactive VR nature scenes and have the potential to improve engagement and emotional regulation in individuals experiencing cognitive decline (Huygelier et al., 2019; Kim et al., 2023; Liao et al., 2020; Niki et al., 2020; Park, 2022; Valtchanov et al., 2010; Zhu et al., 2022). Recent review articles have indicated variability in clinical outcomes based on the level of immersion (i.e., the degree of interaction within the VR nature context), specific characteristics of the VR nature materials presented to participants, and contextual factors that moderate the effectiveness of VR nature-based interventions (Chan et al., 2023; Spano et al., 2023). Additional focused quantitative evaluations of cognitively impaired older adults are required (Borenstein et al., 2009; Higgins et al., 2003; Thompson & Higgins, 2002; Viechtbauer, 2010). Virtual reality (VR)-based nature interventions provide controlled, repeatable, and potentially engaging exposure to restorative natural environments within indoor or supervised care settings (Vass et al., 2022). In this review, VR-based nature interventions refer to simulated natural environments delivered through immersive, semi-immersive, or non-immersive VR systems, including forests, beaches, gardens, green spaces, water landscapes, and other restorative nature scenes. Multimodal programs were considered relevant when nature exposure remained the primary therapeutic component and additional elements, such as music, narration, mindfulness, reminiscence, or breathing guidance, served as supportive components rather than replacing the nature-based exposure. Although prior narrative and broader reviews have examined virtual nature, VR exposure, and psychological outcomes, focused quantitative evidence in cognitively impaired older adults remains limited. Clarifying how intervention duration, immersion level, session frequency, interactivity, delivery setting, and content characteristics influence outcomes is also important for designing standardised, reproducible, and clinically usable VR protocols.

This study aimed to rigorously evaluate the therapeutic effects of nature-based virtual reality (VR) interventions on mental health outcomes in older adults with cognitive impairment. Specifically, this investigation aimed to synthesise pooled effect sizes across a comprehensive range of clinically relevant outcomes, including depression, anxiety, stress/distress, agitation, apathy, mood/affect, loneliness, well-being, quality of life, and sleep quality. The review was designed to estimate the direction and magnitude of effects while also examining variability across studies rather than assuming uniform intervention effects. In addition, the study systematically identified and analysed intervention-related moderators (such as VR immersion level, session duration, frequency, and environmental design features) and participant-related moderators (including age, severity of cognitive impairment, comorbidities, and baseline mental health status) to elucidate the factors influencing the differential efficacy of these interventions. this study sought to provide clinically useful evidence for the development of safer, more standardised, culturally adaptable, and scalable VR-based nature interventions for geriatric cognitive care. the findings are intended to generate robust empirical evidence that informs the development of targeted, personalised, and effective interventions, thereby contributing to the optimisation of mental health support strategies for this vulnerable population and advancing the broader field of digital therapeutics in geriatric care.

## Methodology

### Study design and reporting standards

This systematic review and meta-regression was conducted and reported according to PRISMA 2020 guidelines (Page et al., 2021) and was designed to evaluate the effectiveness, safety, feasibility, and moderators of virtual reality (VR)-based nature interventions for mental health outcomes among older adults with cognitive impairment (See Supplementary File 1). A predefined protocol was registered in the International Prospective Register of Systematic Reviews (PROSPERO: CRD420261285152) with specified objectives, eligibility criteria, and analytical approaches. The protocol specified the review objectives, eligibility criteria, information sources, outcomes, data extraction procedures, risk of bias assessment, certainty of evidence assessment, and statistical analysis plan. The PRISMA flow diagram was prepared using the final reconciled screening counts, and all numbers in the figure were checked against the text to ensure consistency of the data.

### Eligibility criteria

Only randomised controlled trials were included. Eligible participants were older adults aged ≥ 60 years, or study samples with a mean age ≥60 years, with mild cognitive impairment, dementia, Alzheimer’s disease, or another clinically defined cognitive impairment. Studies involving mixed-age samples were eligible only when data for older adults were reported separately or when the mean sample age met the eligibility threshold. Peer-reviewed English-language articles published from January 2000 to December 2025 were considered eligible. The year 2000 was selected because contemporary VR systems and digital therapeutic applications became increasingly relevant after this period. Non-randomised studies and those without clearly defined cognitive impairments were also excluded. Peer-reviewed English-language articles published between 2000 and 2025 were included. When defining eligible intervention populations and clinically relevant therapeutic contexts, prior VR-based intervention studies involving cognitive, behavioural, and psychosocial outcomes in neurologically or psychiatrically affected populations were considered (Khan et al., 2023; Lyu et al., 2024; Maggio et al., 2024; Manuli et al., 2020). Trials were also excluded when the intervention did not include VR, when the virtual environment was not based on nature, or when nature exposure was not the primary therapeutic component.

### Intervention and comparator characteristics

Interventions that qualify are those offered through virtual reality using immersive head-mounted displays, semi-immersive systems, or non-immersive systems and may be passive or interactive. Interventions can also include a guided component (e.g., mindfulness, breathing, narration, music) as long as the primary therapeutic intervention is of a virtual nature exposure. This classification was designed to accommodate the diversity of VR-based therapeutic delivery formats reported in previous intervention studies, including multimodal and rehabilitation-linked VR protocols (Khan et al., 2023; Lyu et al., 2024; Maggio et al., 2024; Manuli et al., 2020). The comparator conditions will consist of standard care, a waitlist/non-treatment control group, a control group that receives non-VR treatment or is exposed to a non-nature virtual experience, or a control for two-dimensional nature exposure.

### Information sources and search strategy

An extensive literature review was conducted using several databases, including MEDLINE/PubMed, Embase via Ovid, PsycINFO, CINAHL, Scopus, Web of Science Core Collection, Cochrane Central Register of Controlled Trials, Cochrane Database of Systematic Reviews, and Google Scholar for supplementary grey-literature screening. Searches were also performed on ClinicalTrials.gov and the World Health Organization’s International Clinical Trials Registry Platform. References from the reference lists and Google Scholar were screened for relevance. Complete database-specific search strategies are provided in Supplementary File 2; authors were contacted when necessary.

### Study selection and data extraction

All identified records were imported into EndNote reference management software for duplicate removal and screening management. Two reviewers independently screened the titles and abstracts, followed by full-text screening of potentially eligible studies. Disagreements were resolved through discussion. Data extraction was performed independently using a piloted extraction form and included the study design, sample size, participant characteristics, diagnostic criteria, intervention characteristics, comparator details, outcome measures, assessment time points, and statistical data necessary for effect size computation and moderator analysis (See Supplementary File 3). Particular attention was paid to the extraction of intervention delivery features, therapeutic components, and assessment tools used in previous VR studies involving cognitive and psychosocial outcomes (Khan et al., 2023; Lyu et al., 2024; Maggio et al., 2024; Manuli et al., 2020).

### Outcomes and timing of measurement

The primary outcomes were mental health outcomes assessed using at least one validated measure of depression, anxiety, stress, distress, agitation, apathy, mood, affect, loneliness, social isolation, psychological well-being, mental health – related quality of life, and sleep quality. Loneliness and social isolation were retained as prespecified mental health – related outcomes because of their established association with morbidity, mortality, and functional decline in older adults (Holt-Lunstad et al., 2015). Secondary outcomes included quality of life, neuropsychiatric symptoms, physiological stress indicators, adherence, attendance, acceptability, and adverse events. Adverse events included cybersickness, dizziness, nausea, headache, visual discomfort, confusion, agitation, fatigue, session discontinuation, and any other VR-related tolerability concern reported by the trial authors. Outcome selection and extraction were also informed by the range of cognitive, emotional, behavioural, and social functioning measures used in previous VR-based intervention studies (Khan et al., 2023; Lyu et al., 2024; Maggio et al., 2024; Manuli et al., 2020). Outcomes were extracted at the immediate post-intervention, short follow-up (≤4 weeks), and longer follow-up (>4 weeks). Immediate post-intervention outcomes were prioritised for the primary meta-analysis, whereas follow-up outcomes were summarised separately when sufficient data were available.

### Risk of bias and certainty of evidence

The risk of bias was independently assessed by two reviewers using the Cochrane Risk of Bias (RoB 2) (Sterne et al., 2019). The domains assessed included the randomisation process, deviations from the intended interventions, missing outcome data, outcome measurement, and selection of the reported results. The certainty of the evidence for primary outcomes was evaluated using the Grading of Recommendations Assessment, Development, and Evaluation (GRADE) approach. Summary of Findings tables were then prepared (Guyatt et al., 2011). Certainty was rated as high, moderate, low, or very low and downgraded for risk of bias, inconsistency, indirectness, imprecision, and publication bias when applicable.

### Data synthesis and meta-analysis

A structured narrative synthesis was performed for all included studies. A meta-analysis was conducted when at least two sufficiently comparable studies reported the same constructs. For continuous outcomes measured using different instruments, standardized mean differences were calculated using Hedges g. For continuous outcomes measured on the same scale, mean differences were calculated when appropriate. For dichotomous outcomes, risk ratios or odds ratios were calculated according to the outcome structure and available data. Risk or odds ratios were calculated for dichotomous outcomes. Random-effects models using restricted maximum likelihood estimation were applied to the data. Effect estimates were reported with 95% confidence intervals. Statistical heterogeneity was quantified using I^2^ and τ^2^, and prediction intervals were calculated where appropriate to show the expected range of true effects in future comparable settings.

### Unit-of-analysis and missing data

For multi-arm trials, the comparator groups were split, or multivariate methods were applied to avoid double-counting (Higgins et al., 2023). Cluster randomised controlled trials were adjusted for clustering using reported intraclass correlation coefficients; when unavailable, coefficients were imputed from similar studies and tested in the sensitivity analyses. For crossover trials, only the first-period data were used when washout or carryover effects were unclear. Missing standard deviations were derived from standard errors, confidence intervals, or *p*-values when possible, or were imputed using pooled values from similar studies with sensitivity analyses.

### Meta-regression, subgroup, and sensitivity analysis

A random-effects meta-regression using restricted maximum likelihood estimation was conducted to explain heterogeneity and identify the predictors of stronger mental health benefits. Analyses were performed using the R statistical software (version 4.3.0) with the metafor package with the metafor package. The prespecified moderators included intervention, participant, and study-level characteristics. Subgroup and sensitivity analyses were conducted according to a pre-specified protocol and reported graphically. Leave-one-out sensitivity analyses were performed to evaluate the influence of individual studies on pooled estimates.

### Publication bias and patient involvement

Publication bias was assessed using funnel plots when at least ten studies were available, supplemented by Egger’s regression test (Egger et al., 1997). Trim-and-fill analysis was performed as an exploratory method to examine the possible influence of missing studies on pooled estimates. Registry records were compared with publications where feasible. Patients or the general public were not involved in the protocol development. The findings from publication-bias analyses were interpreted cautiously because funnel-plot asymmetry may reflect publication bias, small-study effects, clinical heterogeneity, methodological differences, or outcome-reporting variation.

## Results

### Study identification and screening

**Figure 1.**
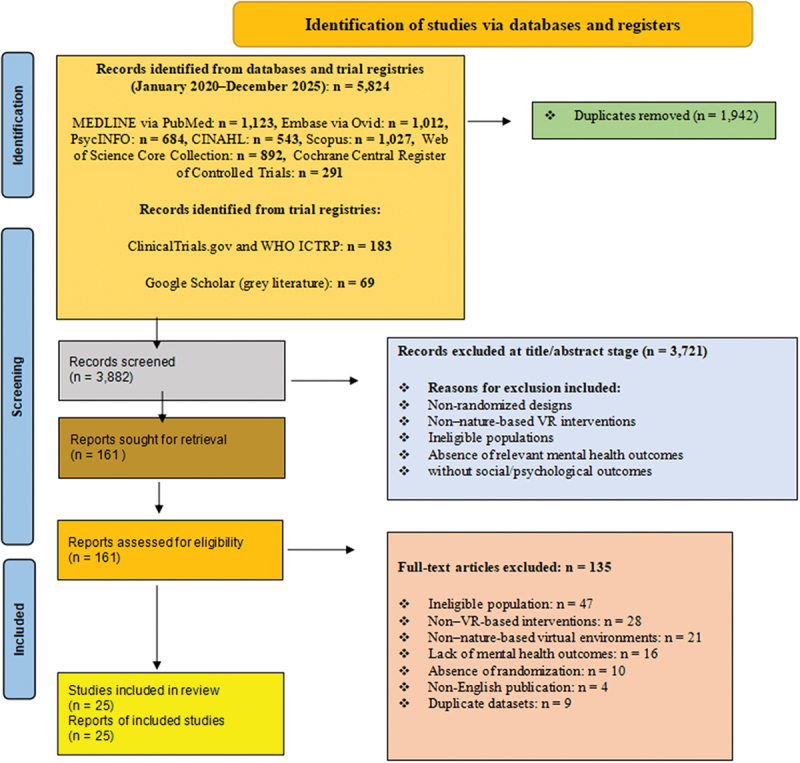
PRISMA flowchart showing the study selection process.

A total of 5,824 records were identified across nine electronic databases and trial registries supplementary sources from January 2000 through December 2025. After removing 1,942 duplicates, 3,882 unique records were screened based on their title and abstract. Of these, 3,721 studies were excluded because of non-randomised designs, non-nature-based VR interventions, ineligible populations, or the absence of relevant mental health outcomes, leaving 161 full-text articles for detailed evaluation. From the review of the full texts, 135 studies were excluded because they were either excluded for ineligible populations (*n* = 47), non-VR-based interventions (*n* = 28), non-nature-based virtual environments (*n* = 21), lack of mental health outcomes (*n* = 16), no randomisation (*n* = 10), published in a language other than English (*n* = 4), or included duplicate datasets (*n* = 10). The remaining 24 randomised controlled trials that met the criteria for randomised controlled trials were eligible for qualitative synthesis and meta-analysis. The overall selection process for all the included studies is illustrated in the PRISMA 2020 flow diagram (See Figure 1).

### Database yield and additional sources

After searching different databases for published studies, the database-specific yields were as follows: From MEDLINE/PubMed, 1, 123 records were retrieved, of which nine studies were included. From Embase/Ovid, 1,012 records were retrieved, of which seven studies were included. From PsycINFO, 684 records were retrieved, resulting in five studies being included. From CINAHL, 543 records were retrieved, of which three studies were included. A total of 1,027 records were retrieved from Scopus, resulting in five studies being included; from the Web of Science Core Collection, 892 records resulted in three studies being included; and from the Cochrane Central Register of Controlled Trials, 291 records yielded two studies. Searching ClinicalTrials.gov and the World Health Organization International Clinical Trials Registry Platform yielded 183 registered trials; one unpublished eligible trial was included via correspondence with the authors. An additional 69 grey literature records were located using Google Scholar; however, no records met the inclusion criteria after the exclusion of duplicates. A manual search of the reference lists and citation tracking of the published studies included yielded two additional eligible registered trials. Database-specific included-study counts were not mutually exclusive because some eligible studies were indexed in more than one source.

### Characteristics of included studies

The final sample comprised 24 randomised controlled trials including 1,534 participants. Participants were older adults aged ≥ 60 years, or study samples with a mean age ≥ 60 years, with clinically defined cognitive impairment, mild cognitive impairment, Alzheimer’s disease, or dementia. The included studies evaluated a range of VR-based nature interventions, including immersive virtual forest walks, seaside and beach simulations, virtual gardens, and green space relaxation experiences, delivered in community, home-based, or long-term care settings, and compared with non-VR controls or alternative interventions. A supplementary intervention-characteristics table summarises VR device type, immersion level, natural environment content, interactivity, guidance components, session duration, session frequency, total intervention duration, comparator condition, delivery setting, outcome measures, and follow-up timing. These trials collectively formed the evidence base for the meta-analysis and meta-regression assessing the effects of VR-based nature interventions on depression, anxiety, stress, agitation, apathy, loneliness, well-being, and quality of life outcomes, with detailed study characteristics provided. The key characteristics of the included studies and database-specific contributions are presented in Table 1.

**Table 1.** Study characteristics and PICO-related outcome statistics of the included studies.

Study	Population Description	Inclusion Criteria	Exclusion Criteria	Intervention Characteristics	Comparator Details	Outcome Measures	PICO
Buonocore et al. (2025)	Patients with Parkinson’s disease – related mild cognitive impairment	Idiopathic PD; PD-MCI; age 50–85; stable medication	Other neurological disorders; psychiatric illness; sensory deficits	Therapist-guided home-based VRRS-Home Kit cognitive stimulation; 4 weeks, 20 sessions, ~45 min/session; multi-domain	In-person outpatient cognitive stimulation; paper-and-pencil; same duration	MMSE, MoCA, RAVLT, Digit Span, TMT-A/B, FAB, BADA, FAS, ADL, IADL, STAI, CRIq	P: PD-MCI adults; I: VR-based tele-cognitive stimulation; C: in-person stimulation; O: cognitive/emotional outcomes
Latorre et al. (2025)	Individuals with mild – moderate dementia in structured care	Diagnosis of dementia; capacity to engage; consent	Seizures, vertigo, epilepsy, hallucinations, severe behavioural disorders	Immersive VR nature exposure using Nature Treks VR and personalized 360° videos; Oculus Go; 12 sessions per phase, 15 min/session	Tablet-based cognitive stimulation (object matching, word search); same dose	Heart rate; I-PANAS-SF; STAI-r; behavioural observation; subjective response questionnaire	P: Dementia patients; I: VR nature; C: Tablet activities; O: HR, affect, anxiety
Del Din et al. (2020)	Older adult fallers with or without MCI or Parkinson’s disease	Age 60–90; ≥2 falls in past 6 months; ambulatory; stable medication	Major neurological or medical instability	Treadmill training plus virtual reality (TT+VR); 6 weeks; 3 sessions/week; 40 min/session; non-immersive VR	Active comparator: treadmill training alone (TT) with same dose	Falls Rate to Activity index; number of falls; walking activity parameters	P: Older fallers; I: TT+VR; C: TT; O: FRA index and falls
Saredakis et al. (2021)	Older adults ≥ 65 years living in residential aged care with mild – moderate cognitive impairment	≥65 years; English speaking; Psychogeriatric Assessment Scale ≤ 15; able to consent; VR group able to tolerate HMD	Severe cognitive impairment; confusion/distress risk; conditions preventing assessment; intolerance of HMD	Reminiscence therapy using immersive VR via head-mounted display (Oculus Quest); 3 individual sessions; ~20 min/session over ~2 weeks; personalized 360° video & Google Street View content	Active control: reminiscence via laptop + optional physical items; Passive control: usual care, no intervention	Primary: AES (apathy); Secondary: ACE-III (cognition), GDS (depression); Exploratory: QOL-AD, Three-Item Loneliness Scale; Safety: Simulator Sickness Questionnaire (SSQ); Session Record	P: Residential aged care older adults with apathy; I: VR-based reminiscence therapy; C: Laptop reminiscence or usual care; O: Apathy, cognition, depression, QoL
Fiorenzato et al. (2025)	Adults with Parkinson’s disease and mild cognitive impairment; parallel healthy ageing cohort	PD age-matched adults; PD-MCI per MDS criteria; ability to complete home iVR training; informed consent	Severe cognitive impairment/dementia; MMSE < 26 for HC; inability to complete VR or assessments	EF-training (iVR CT): immersive VR supermarket environment via Oculus Go; telemedicine-monitored; 3 sessions/week, 30 min/session, total 20 sessions over 4 weeks; tasks: Planning (Zoo Map – based), Shifting (alternating category selection), Updating (n-backward recall); adaptive difficulty; remote monitoring	Active Placebo (AP): same VR environment and schedule but low-cognitive-load tasks (placebo-Planning, placebo-Shifting, placebo-Updating); no EF challenge	Primary: Prospective Memory (MIST total, time-based, event-based, action-response, verbal-response, 24-h task, recognition); Executive Functions (Zoo Map, TOL, TMT, MCST, Stroop, Digit Span, Corsi, TAP). Secondary: PRMQ, PMQ, RMQ; BDI-II, STAI-Y1/Y2, AES; ADL, IADL, PD-CFRS; PDQ-39, OPQOL-35. Feasibility: SUS, nausea, dizziness, fatigue	P: PD-MCI adults. I: iVR EF-training. C: Active placebo VR. O: PM, EF, QoL, neuropsychiatric outcomes
Walden and Feliciano (2021)	Older adult females with dementia in LTC	≥65 years; dementia diagnosis; female; English-speaking; resident ≥ 2 weeks; no active psychosis; POA consent	Active psychosis; inability to assent	VR nature scenes via Oculus Go HMD using Guided Meditation VR; brief antecedent intervention; seated use; duration varied by participant	Baseline and withdrawal phases served as control conditions	CMAI agitation behaviours (pacing, repetitive vocalizations), SLUMS	P: dementia residents; I: VR nature scenes; C: baseline/no VR; O: agitation reduction
Park et al. (2020)	Community-dwelling older adults with amnestic mild cognitive impairment	Age > 60; subjective memory complaint; objective memory impairment; MMSE-K ≥ 24; intact ADL	Dementia diagnosis; neurological/psychiatric disorders; moderate – severe depression; sensory impairments; recent cognitive training	Virtual reality – based spatial cognitive training (VR-SCT); 24 sessions; 45 min/session; 3 days/week for 8 weeks; all centric navigation using boundary cues; joystick-controlled desktop VR; Unity engine; gem-finding navigation task; Euclidean distance feedback	Waitlist control; no intervention during study; received same VR-SCT after trial completion	WAIS-BDT (spatial cognition); Seoul Verbal Learning Test (SVLT recall and recognition); Euclidean distance error during training	P: Older adults with amnestic MCI; I: VR-based spatial cognitive training; C: Waitlist control; O: Spatial cognition, episodic memory
Kang et al. (2021)	Older adults (>60 years) with subjective cognitive decline or mild cognitive impairment	Age > 60; SCD or MCI; MMSE ≥ 20; intact ADL; consent capacity	Major psychiatric disorder; dementia; neurodegenerative disease; MRI abnormalities; inability to use VR	Fully immersive VR multidomain cognitive training; Oculus Rift CV1; 8 sessions (2/week for 1 month); 20–30 min/session; tasks covering attention, executive function, memory, visuospatial orientation; difficulty levels 1–4	Usual care only (pharmacotherapy such as choline alfoscerate or cholinesterase inhibitors)	Primary: RCFT copy task. Secondary: MMSE, Digit Span, TMT-A/B, K-BNT, SVLT, COWAT, Stroop, GDS, AES, PANAS, QoL-AD, rsfMRI connectivity, SSQ	P: Predementia older adults; I: Fully immersive VR cognitive training; C: Usual care; O: Visuospatial function, psychiatric symptoms, brain connectivity
Park et al. (2020)	Community-dwelling older adults with amnestic mild cognitive impairment	Age > 60; subjective memory complaint; objective memory impairment; MMSE-K ≥ 24; intact ADL	Dementia diagnosis; neurological/psychiatric disorders; moderate – severe depression; sensory impairments; recent cognitive training	Virtual reality – based spatial cognitive training (VR-SCT); 24 sessions; 45 min/session; 3 days/week for 8 weeks; all centric navigation using boundary cues; joystick-controlled desktop VR; Unity engine; gem-finding navigation task; Euclidean distance feedback	Waitlist control; no intervention during study; received same VR-SCT after trial completion	WAIS-BDT (spatial cognition); Seoul Verbal Learning Test (SVLT recall and recognition); Euclidean distance error during training	P: Older adults with amnestic MCI; I: VR-based spatial cognitive training; C: Waitlist control; O: Spatial cognition, episodic memory
Torpil et al. (2021)	Older adults aged 65–75 years with MCI	Age 65–75; MCI diagnosis; comprehension ability	Chronic disease affecting cognition; sensory impairments; concurrent rehabilitation	CR+VR: Conventional cognitive rehabilitation plus Kinect-based non-immersive VR games; 45 min sessions, twice weekly for 12 weeks; games: Boxing Trainer, Jet Run, Super kick, Air Challenge	Control: Conventional cognitive rehabilitation only, same duration/frequency	LOTCA-G cognitive domains: orientation, visual perception, spatial perception, motor praxis, visuomotor organization, thinking operation, memory, attention/concentration	P: Older adults with MCI; I: CR+VR; C: CR only; O: Cognitive function improvement
Sasaninezhad et al., 2024	Adults with MCI (transitional stage between normal ageing and dementia)	Diagnosed MCI; adults; consent provided	Dementia; neurological disease; non-eligibility for cognitive testing	VR-based cognitive rehabilitation targeting cognitive flexibility, verbal & visual working memory, IADLs; culturally contextualized Iranian VR; multiple VR cognitive tasks	Control group received no VR cognitive rehabilitation	IADLs, WCST-64 (cognitive flexibility), Digit Span, Symbol Span, anxiety, depression	P: Adults with MCI; I: VR cognitive rehabilitation; C: Control (no VR); O: Cognitive, emotional & functional outcomes
Chan et al. (2010)	Chinese older adults with chronic schizophrenia living in institutional care	Age ≥ 60; schizophrenia > 20 years; MMSE 15–23; independent ambulation; no upper limb restriction; written consent	Mental retardation, dementia, brain injury, other diseases compromising cognition	Adapted VR cognitive training using IREX Gesture Xtreme system; two VR activities (Ball and Bird sessions 1–5; Shark Bait sessions 6–10); 10 sessions total; each 15 minutes; twice weekly; difficulty progressively increased; delivered by occupational therapist	Usual programs in residential facility; no VR during study period	Cognistat (total + subscales), Volitional Questionnaire (VQ), Simulator Sickness Questionnaire (SSQ)	P: Older adults with chronic schizophrenia; I: VR cognitive training; C: Usual care; O: Cognitive function, volition, cyber sickness
Oliveira et al. (2021)	Older adults with major neurocognitive disorder due to AD	>65 years; AD diagnosis; Portuguese speaking; consent	Psychiatric disorders; severe language or sensory-motor impairment	Non-immersive VR cognitive stimulation using Systemic Lisbon Battery; 12 sessions; 45 min; ~9 h total; IADL-based tasks	Treatment as usual at residential care units	Executive function (FAB, TMT); Global cognition (MMSE, CDT); IADL; Depression (GDS-15); Dementia rating (CDR)	P: AD dementia patients; I: VR cognitive stimulation; C: TAU; O: cognition, function
Jeong et al. (2024)	Adults with cognitive impairment after ABI	Age > 19; ABI confirmed by imaging; adequate motor strength	Severe aphasia; psychiatric disorder; severe depression; uncontrolled illness	VR-MAT: 4-level virtual drumming attention training, 30 min/session, 5x/week, 8 weeks	Conventional cognitive training (card sorting, computerized tasks)	TMT, TMT-B&W, CDR-SB, GDS, MMSE, AST, SST, SWM, VR-MAT accuracy	P: ABI adults I: VR-MAT C: CCT O: Attention & executive function
Hajebrahimi et al. (2022)	Adults with Parkinson’s disease	PD diagnosis, ≥50 years, able to walk 5 min unassisted, stable medication	Dementia, major depression, MRI contraindications, DBS, severe comorbidities	Virtual Reality-based Exergaming using Nintendo Wii Fit Plus; 60 min/session, 3x/week, 4 weeks (12 sessions)	Exercise Therapy (balance, gait, strengthening exercises)	UPDRS-III, BBS, TUG, 6MWT, MoCA, VMPT, Stroop, BNT, PDQ-39, rs-fMRI	P: PD patients; I: VR-based exergaming; C: Exercise therapy; O: Cognitive and motor function
Rogers et al. (2019)	Adults with sub-acute stroke and upper-limb dysfunction	≥18 years; unilateral stroke; English communication; able to follow instructions; able to sit unsupported	Prior neurological/psychiatric disorder; severe visual impairment; age < 18	Elements virtual rehabilitation: 12 sessions over 4 weeks; 3×/week, 30–40 min/session; goal-directed + exploratory upper-limb VR tasks with tangible interfaces; augmented visual & auditory feedback; combined with usual occupational & physiotherapy	Treatment as Usual (TAU) only: ~3 h/day conventional occupational + physiotherapy	Primary: Box and Blocks Test (BBT); Secondary: MoCA, Cog State GMLT, Set Shift Task, Neurobehavioral Functioning Inventory (NFI)	P: sub-acute stroke adults; I: Elements VR + TAU; C: TAU alone; O: motor, cognitive, functional recovery
Kwan et al. (2024)	Older adults with cognitive frailty (MCI + physical frailty)	≥60 years; community dwelling; MoCA < 26; Fried frailty > 0; ambulatory	Dementia diagnosis; mobility restriction (FAC < 7)	VR motor-cognitive training: cycling ergometer + HTC Vive VR; 16 sessions, 1 h/session, 2x/week, 8 weeks; gamified IADL themes	Usual care at community centres; no VR during intervention period	MoCA, Fried Frailty Phenotype, Timed Up and Go, Digit Span Forward/Backward, Stroop Test, Trail Making Test	P: cognitive frailty; I: VR motor-cognitive training; C: usual care; O: cognition, frailty, mobility
Choi and Lee (2019)	Older adults with mild cognitive impairment	Age ≥ 65; MoCA < 26; able to communicate; commit to 6 weeks	Neurological/musculoskeletal impairment; dementia; depression; visual deficits; inability to sit ≥ 20 min	Virtual Kayak Paddling exercise: 60 min/session, twice/week for 6 weeks; warm-up 10 min, VKP 40 min, cool down 10 min; 1 kg paddle; VR video kayaking with directional arrows; seated on foam	Home exercise program twice/week for 6 weeks (William flexion, curl-ups, leg lifts, trunk extensions)	Postural balance (OLS, TUG, FRT, BBS, FSST), muscle performance (arm curl, handgrip), cognitive function (MoCA, GPCOG)	P: older adults with MCI; I: VKP exercise; C: home exercise; O: balance, strength, cognition
Man et al. (2013)	Adults with traumatic brain injury undergoing vocational rehabilitation	Age 18–55; Modified Barthel Index ≥ 90; MMSE ≥ 18; TONI-III IQ ≥ 90; medically stable	Severe visual impairment; severe motor impairment; lack of awareness deficit; other neurological disease	Artificial intelligent 3-D virtual reality vocational problem-solving training; 12 sessions; 20–25 min/session; clerical-type vocational simulations; adaptive AI difficulty	Psycho-educational vocational training program (manual-based, therapist-led)	Wisconsin Card Sorting Test (WCST); Tower of London Test; Vocational Cognitive Rating Scale (VCRS); employment status; self-efficacy	P: adults with TBI; I: AI-based VR vocational training; C: psycho-educational training; O: executive function, vocational cognition, employment
Tang et al. (2024)	Late middle-aged and older adults (50–75 years), normal BMI, cognitively intact	Age 50–75; BMI 18.5–24.9; MMSE ≥ 24; Able to cycle; Consent	OCD or anxiety disorder; MMSE < 24; Claustrophobia; Psychiatric disorder	20-min cycling in CAVE immersive VR; Moderate intensity (50–60% HRmax); HTDS: 36–60% tree density; MTDS: 20–35% tree density; EEG recorded via FP1, FP2, T3, T4; Alpha band 8–13 Hz; Sampling 256 Hz; Band-pass 0.2–35 Hz	Control: 20-min cycling facing white wall in VE lab (non-VR nature exposure)	Primary outcome: EEG alpha frequency band power (μV). Secondary: Satisfaction (1–5 scale) and PACES enjoyment scale (1–5).	P: Adults aged 50–75 years I: 20-min cycling in VR with varying tree density. C: Cycling while facing a white wall. O: EEG alpha power, enjoyment, and satisfaction.
Lee et al. (2015)	Community-dwelling older women ≥ 65 years	Female ≥ 65; capable of communication; passed Par-Q & HHQ; able to participate physically	Cardiovascular disease; musculoskeletal disorder; visual/hearing impairment; physical activity limitation; communication limitation	12 weeks; 3x/week; 30 min/session (10 min VR cognitive; 10 min motion tracking physical RPE 11–13; 10 min touchscreen cognitive). Progressive difficulty. Independent participation.	Control: maintained the usual lifestyle (no structured digital intervention).	Primary: Cognitive function (MoCA), Depression (SGDS-K). Secondary: Body composition (weight, lean mass, fat mass, fat %, BMI), Quality of life (8 domains WHOQOL-BREF).	P: Older women ≥ 65. I: 12-week digital VR + motion-tracking + touchscreen training. C: Usual lifestyle. O: Cognitive function, depression (primary); body composition & QoL (secondary).
Primavera et al. (2024)	Adults aged 58–75 with bipolar disorder; cognitively borderline; depressive symptoms near severe cut-off	Age 58–75; DSM-IV bipolar disorder; informed consent	Acute depression/mania; epilepsy; severe eye disease	Treatment as usual (TAU) + VR Cognitive Remediation (CEREBRUM v3.0.1); 2 weekly sessions × 3 months; 52 VR exercises simulating daily life; clinician-adapted difficulty; multidisciplinary team	TAU only (psychiatric visits + pharmacotherapy)	15 cognitive tests: Rey – Osterrieth; Rey Words Immediate/Delayed; Story Recall; Digit Span Forward/Backward; TMT-A/B; Matrix; Verbal Fluency (phonological/semantic); Stroop; DSST; FAB; CET. Depression: PHQ-9	P: Older adults (58–75 years) with bipolar disorder. I: TAU + VR cognitive remediation (3 months). C: TAU only. O: Cognitive performance & depressive symptoms (PHQ-9).
Thapa et al. (2020)	Community-dwelling older adults with MCI (55–85 years)	Age 55–85; MCI diagnosis by specialist; Consent	Dementia diagnosis; past psychiatric/neurological disorder; non-consent	8 weeks; 3×/week; 100 min/session; 24 sessions total; Fully immersive Oculus Quest; 4 VR games (Juice Making, Crow Shooting, Fireworks number memory, Love House object memory); Included eye massage/stretching intervals; EEG recorded with 19-channel Cognionics Quick-20; Sampling 500 Hz; Band-pass 0.53–120 Hz; ICA artefact removal; TBR, TAR, DAR analysed.	Weekly general health education (eight sessions; 30–50 min) led by health professionals; nutrition and exercise advice; no VR exposure.	Primary: Cognitive (MMSE, TMT A/B, SDST). EEG: Theta power; Theta/Beta Ratio (TBR). Secondary outcomes included grip strength, gait speed (4 m), and 8-ft Up & Go.	P: Older adults (55–85 years) with MCI. I: 8-week fully immersive VR cognitive training. C: Health education only. O: Cognitive tests (MMSE, TMT, and SDST), EEG power ratios, and physical function.
Lin et al. (2020)	Institutionalized older adults ≥ 65 years in long-term care facilities	≥65 years; long-term resident; able to understand instructions; joystick operation ability	Severe psychiatric disorder; dementia; major sensory impairment; stroke; Parkinson disease	9 weeks; 18 sessions; 2×/week; 1 hour/session; Combination of 3D VR horticultural modules (5–10 min each; 3 VR sets shared) + hands-on horticultural therapy; Facilitated by 2 certified horticultural therapists + trained graduates (16-hour workshop); VR helmets & joystick; Additional voluntary VR practice allowed	No intervention; routine activities; no similar program during intervention and follow-up	Health status (CHQ-12); Meaning in Life (9 items); Perceived Mattering (5 items); Loneliness (ULS-6, reverse scored); Depression (GDS-15, reverse scored)	P: Institutionalized adult aged ≥ 65 years I: 9-week combined 3D VR + horticultural therapy. C: Routine care. O: Health status, meaning of life, perceived mattering, loneliness, and depression.
Cheng et al. (2022)	Institutionalized older adults ≥ 65 years	≥65 years; institutional resident; able to operate VR device; cognitively intact; consent provided	Severe psychiatric disorder; dementia; major visual/hearing impairment; stroke; Parkinson disease	9-week program; 18 sessions; 2×/week; 1 hour/session; Combination of immersive 3D VR aromatherapy modules (5–10 min exposure per rotation; 3 VR devices) + hands-on aromatherapy product creation; Facilitated by certified aroma therapists + trained assistants; Additional voluntary VR access allowed	Routine activities only; no aromatherapy or VR exposure during intervention/follow-up	Health status (CHQ-12); Happiness scale; Perceived stress; Loneliness (ULS-6, reverse scored); Depression (GDS-15, reverse scored)	P: Institutionalized adults aged ≥ 65 years. I: 9-week 3D VR + aromatherapy program. C: Routine care. O: Health status, happiness, stress, loneliness, and depression.
Lee et al. (2015)	Community-dwelling older women ≥ 65 years	≥65 years; MMSE > 23; independent standing	Vision/hearing impairment; orthopedic/neurological surgery history; dementia; headaches/dizziness	IFVR: 60 min/session, 3×/week, 8 weeks; 5-min warm-up/cool-down; Kinect + Xbox 360; “Zen Energy” Tai-chi – based movements; 3D avatar with real-time visual & auditory feedback; 4 major lower-body balance motions; individualized scheduling; unsupervised except emergency standby	Group-based exercise: 60 min/session, 3×/week, 8 weeks; physiotherapist-led; postural, balance, lower extremity coordination & strength exercises; auditory group feedback	Primary: HRQoL (SF-36:8 subscales + PCS & MCS). Secondary: Physical fitness 30SCST, 2MSand and T, 8FUGT. Exercise attendance rate	P: Older women ≥ 65 years I: 8-week individualized feedback VR exercise program. C: Group-based physiotherapist-led exercise. O: HRQoL (SF-36) and physical fitness.
Monteiro-Junior et al. (2017)	Institutionalized older adults ≥ 60 years	≥60 years; independent ambulation; medical clearance; able to understand commands	Severe cardiorespiratory impairment; acute musculoskeletal injury; delirium; moderate-to-severe dementia	6 exergames exercises; 30–45 min/session; twice weekly; 12–16 sessions; HR monitored; low – moderate intensity (50–71% HRmax); VR-based exergames	Same 6 exercises without VR stimulation; identical duration/frequency	Primary: Global cognition (MMSE); executive function (TMT, FMT route, and recall); memory (DSF and DSB); and semantic memory (VFT). Secondary: CST; AC; 8ftUG; gait kinematics (SL, SV, MS); depressive symptoms (GDS); fear of falling (FES-I)	P: Institutionalized adults aged ≥ 60 years I: VR-based exergame exercise 12–16 sessions. C: Same exercises without VR headset. O: Cognition, physical function, depression, and fear of falling.

## Neuropsychiatric and safety outcomes

**Figure 2.**
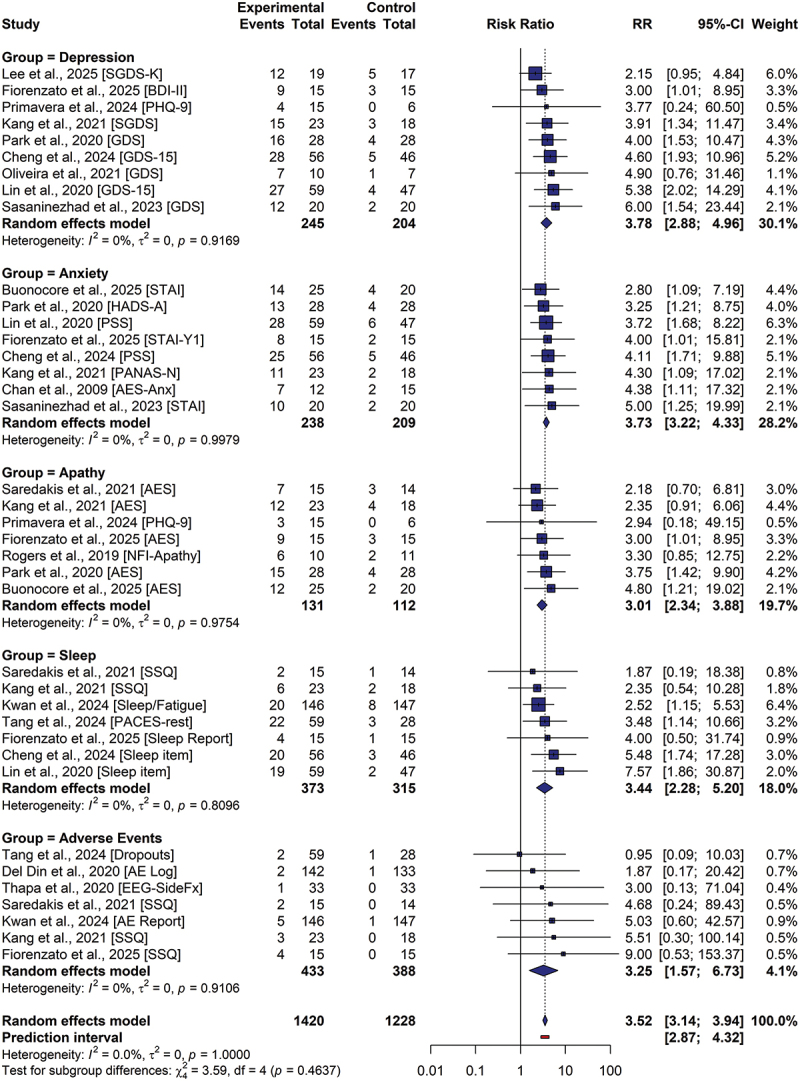
Forest plots of dichotomous clinical outcomes.

Depression outcomes favoured the intervention across nine trials (experimental, *n* = 245; control, *n* = 204), yielding a pooled risk ratio (RR) of 3.78 (95% CI, 2.88–4.96; weight, 30.1%; I^2^ = 0%) (See Figure 2A; Table 2). Individual RRs ranged from 2.15 (95% CI, 0.95–4.84) to 6.00 (95% CI, 1.54–23.44). Anxiety reduction across eight trials (*n* = 238 vs. 209) showed a pooled RR of 3.73 (95% CI, 3.22–4.33; weight, 28.2%; I^2^ = 0%), with study-specific RRs between 2.80 (95% CI, 1.09–7.19) and 5.00 (95% CI, 1.25–19.99) (See Figure 2B; Table 2). Apathy improved in seven trials (*n* = 131 vs. 112) with a pooled RR of 3.01 (95% CI, 2.34–3.88; weight, 19.7%; I^2^ = 0%) (See Figure 2C; Table 2). Sleep quality improved across seven trials (*n* = 373 vs. 315), with a pooled RR of 3.44 (95% CI, 2.28–5.20; weight, 18.0%; I^2^ = 0%) (See Figure 2D; Table 2). Adverse events were reported in seven trials (*n* = 433 vs. 388) with a pooled RR of 3.25 (95% CI, 1.57–6.73; weight, 4.1%; I^2^ = 0%) (See Figure 2E; Table 2). The study-specific estimates ranged from 0.95 (95% CI, 0.09–10.03) to 9.00 (95% CI, 0.53–153.37). Adverse-event findings were interpreted separately from clinical improvement outcomes because a higher RR indicated more frequent reporting of VR-related events, not greater benefit. Across trials, reported events were generally described as transient VR-related symptoms, such as dizziness, nausea, visual discomfort, headache, confusion, or session intolerance, although definitions and reporting thresholds varied. The overall pooled RR across 38 comparisons (*n* = 1420 vs. 1228) was 3.52 (95% CI, 3.14–3.94), with a prediction interval of 2.87–4.32 and no heterogeneity (I^2^ = 0%; P for subgroup difference = 0.46).

A. Depression response rate. B. Anxiety response rate. C. Apathy improvement. D. Sleep improvement. E. Adverse events.

**Table 2.** Pooled clinical outcomes and safety.

Outcome Domain	No. of Trials	Experimental (n)	Control (n)	Pooled Risk Ratio (95% CI)	Weight, %	I^2^, %
Depression Improvement	9	245	204	3.78 (2.88–4.96)	30.1	0
Anxiety Reduction	8	238	209	3.73 (3.22–4.33)	28.2	0
Apathy Reduction	7	131	112	3.01 (2.34–3.88)	19.7	0
Sleep Quality Improvement	7	373	315	3.44 (2.28–5.20)	18.0	0
Adverse Events	7	433	388	3.25 (1.57–6.73)	4.1	0
Overall Effect	38	1420	1228	3.52 (3.14–3.94)	100.0	0
**Prediction interval (overall)**: 2.87–4.32 **Test for subgroup differences**: χ^2^_4_ = 3.59; *p* = .46						

*Note*: All pooled estimates were derived from random-effects models, with no observed statistical heterogeneity across domains (I^2^ = 0% for all comparisons).

## Standardised neuropsychiatric outcomes

**Figure 3.**
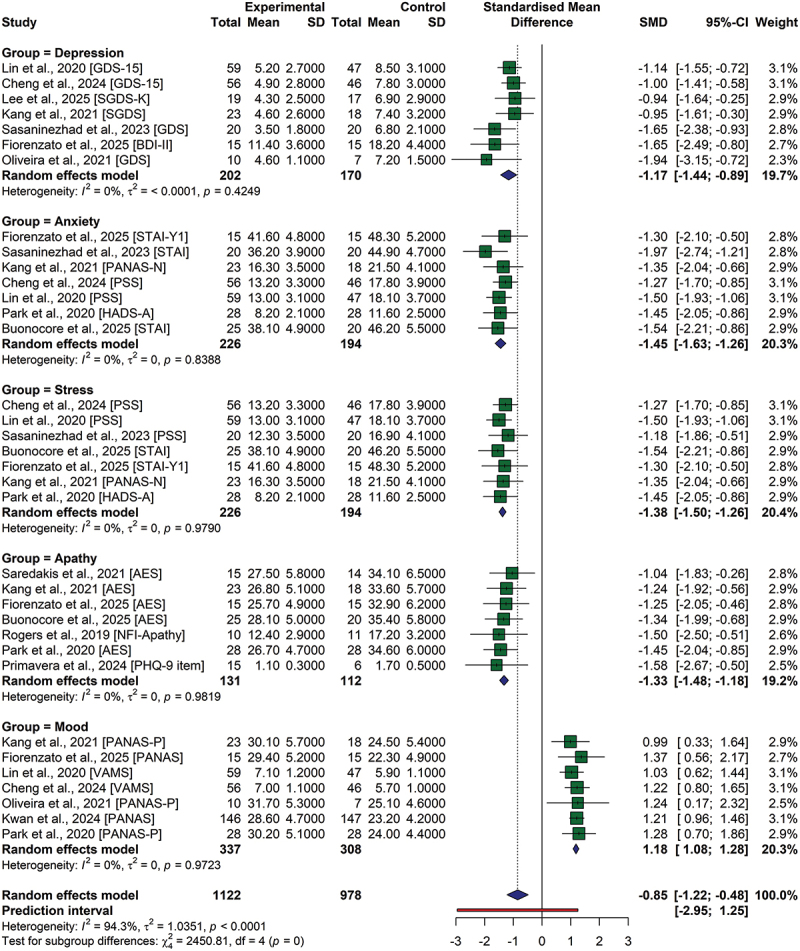
Forest plots of continuous neuropsychiatric symptom outcomes.

Depression scores across the seven trials (*n* = 202 experimental; *n* = 170 control) showed a pooled standardized mean difference (SMD) of −1.17 (95% CI, −1.44 to −0.89; I^2^ = 0%) (See Figure 3A; Table 3). Individual estimates ranged from −0.94 (95% CI, −1.64 to −0.25) to −1.94 (95% CI, −3.15 to −0.72). Anxiety outcomes from the seven trials (*n* = 226 vs. 194) yielded a pooled SMD of −1.45 (95% CI, −1.63 to −1.26; I^2^ = 0%) (See Figure 3B; Table 3), with study-specific effects between −1.27 (95% CI, −1.70 to −0.85) and −1.97 (95% CI, −2.74 to −1.21). Stress or distress scores (seven trials; *n* = 226 vs. 194) showed a pooled SMD of −1.38 (95% CI, −1.50 to −1.26; I^2^ = 0%) (See Figure 3C; Table 3). Apathy scores across the seven trials (*n* = 131 vs. 112) produced a pooled SMD of −1.33 (95% CI, −1.48 to −1.18; I^2^ = 0%) (See Figure 3D; Table 3). Mood and positive affect outcomes from seven trials (*n* = 337 vs. 308) favoured the intervention, with a pooled SMD of 1.18 (95% CI, 1.08 to 1.28; I^2^ = 0%) (See Figure 3E; Table 3). The overall pooled SMD across 35 comparisons (*n* = 1122 vs. 978) was −0.85 (95% CI, −1.22 to −0.48), with substantial heterogeneity (I^2^ = 94.3%; τ^2^ = 1.0351; *p* < .001) and a prediction interval of −2.95 to 1.25. The overall estimate combined several clinically distinct symptom and affect domains, and because heterogeneity was substantial, domain-specific pooled estimates were emphasised over the combined overall effect.

A. Depression scores. B. Anxiety scores. C. Stress/distress scores. D. Apathy scores. E. Mood/positive affects.

**Table 3.** Standardized mean differences for neuropsychiatric outcomes.

Outcome Domain	No. of Trials	Experimental (n)	Control (n)	Pooled SMD (95% CI)	Weight, %	I^2^, %
Depression Scores	7	202	170	−1.17 (−1.44 to −0.89)	19.7	0
Anxiety Scores	7	226	194	−1.45 (−1.63 to −1.26)	20.3	0
Stress/Distress Scores	7	226	194	−1.38 (−1.50 to −1.26)	20.4	0
Apathy Scores	7	131	112	−1.33 (−1.48 to −1.18)	19.2	0
Mood / Positive Affect Scores	7	337	308	1.18 (1.08 to 1.28)	20.3	0
Overall Effect	35	1122	978	−0.85 (−1.22 to −0.48)	100.0	94.3
**Between-study heterogeneity (overall)**: τ^2^ = 1.0351; *p* < .001 **Prediction interval (overall)**: −2.95 to 1.25 **Test for subgroup differences**: χ^2^_4_ = 2450.81; *p* < .001						

*Note*: Negative SMD values favoured interventions for symptom reduction, whereas positive SMD values favoured interventions for mood/positive affect improvement.

## Psychosocial and quality-of-life outcomes

**Figure 4.**
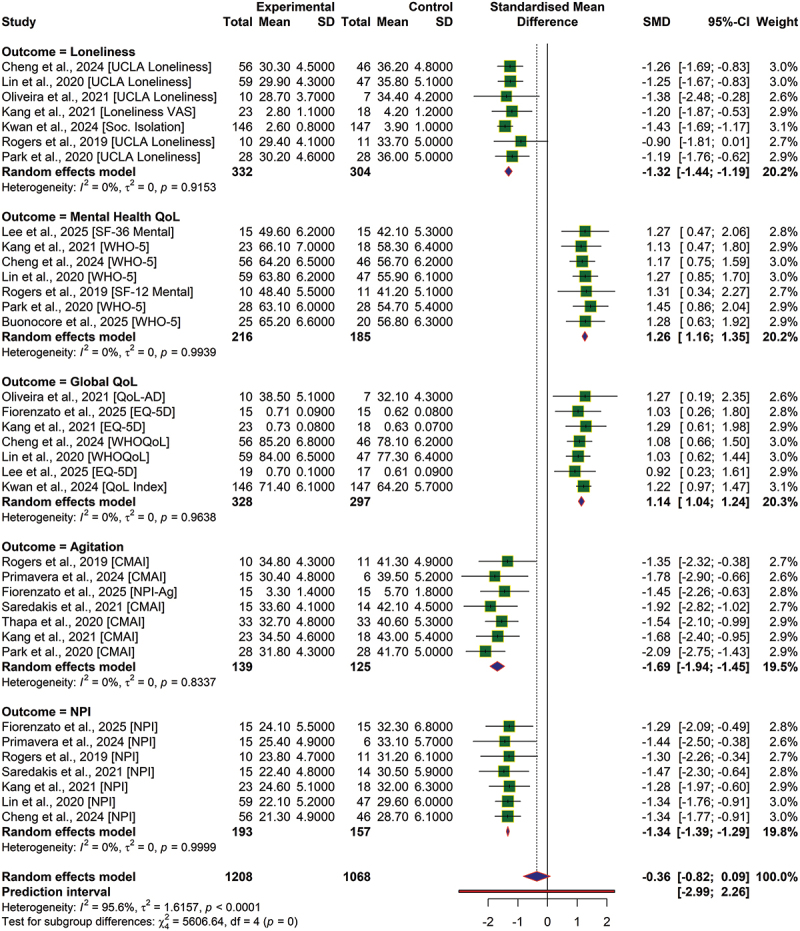
Forest plots of psychosocial and quality-of-life outcomes.

Loneliness across the seven trials (*n* = 332 experimental; *n* = 304 control) yielded a pooled standardized mean difference (SMD) of −1.32 (95% CI, −1.44 to −1.19; I^2^ = 0%) (See Figure 4A; Table 4), with individual effects ranging from −0.90 to −1.43. Mental health – related quality of life from seven trials (*n* = 216 vs. 185) showed improvement, with a pooled SMD of 1.26 (95% CI, 1.16 to 1.35; I^2^ = 0%) (See Figure 4B; Table 4). The pooled SMD of global quality of life (seven studies (*n* = 328 vs. 297). The pooled SMD (*n* = 7) (SMD = 1.14 [95% CI = 1.04–1.24; I ^2^ = 0 %] (See Figure 4C and Table 4). Agitation (seven studies (*n* = 139 vs. 125) score was pooled SMD of −1.69 (95% CI = −1.94–1.45; I^2^ = 0%) (See Figure 4D and Table 4). Neuropsychiatric Inventory (7 studies (*n* = 193 vs. 157) showed a pooled SMD of-1.34 (95% CI = −1.39 to −1.29; I^2^ = 0%) (See Figure 4E: Table 4). The overall pooled SMD across 35 comparisons (*n* = 1208 vs. 1068) = − 0.36; 95% CI= (− 0.82 to 0.09); significant heterogeneity (I^2^ = 95.60%; nucleotide-type II variation coefficient (τ^2^) =1.6157; *p* < .001) with prediction interval = − 2.99–2.26. This combined estimate crossed the null and included outcomes scored in different directions; therefore, the individual domain estimates for loneliness, quality of life, agitation, and neuropsychiatric symptoms provide a clearer interpretation.

A. Loneliness. B. Mental health-related quality of life. C. Global quality of life. D. Agitation.E.Neuropsychiatric Inventory scores.

**Table 4.** Psychosocial and quality-of-life outcomes.

Outcome Domain	No. of Trials	Experimental (n)	Control (n)	Pooled SMD (95% CI)	Weight, %	I^2^, %
Loneliness	7	332	304	−1.32 (−1.44 to −1.19)	20.2	0
Mental Health – Related QoL	7	216	185	1.26 (1.16 to 1.35)	20.2	0
Global Quality of Life	7	328	297	1.14 (1.04 to 1.24)	20.3	0
Agitation (CMAI/NPI-Ag)	7	139	125	−1.69 (−1.94 to −1.45)	19.5	0
Neuropsychiatric Inventory (NPI)	7	193	157	−1.34 (−1.39 to −1.29)	19.8	0
Overall Effect	35	1208	1068	−0.36 (−0.82 to 0.09)	100.0	95.6
**Between-study heterogeneity (overall)**: τ^2^ = 1.6157; *p* < .001 **Prediction interval (overall)**: −2.99 to 2.26 **Test for subgroup differences**: χ^2^_4_ = 5606.64; *p* < .001						

*Note*: Negative SMD values reflected symptom reduction, and positive SMD values reflected improvement in quality-of-life domains.

## Engagement outcomes and physiological stress markers

**Figure 5.**
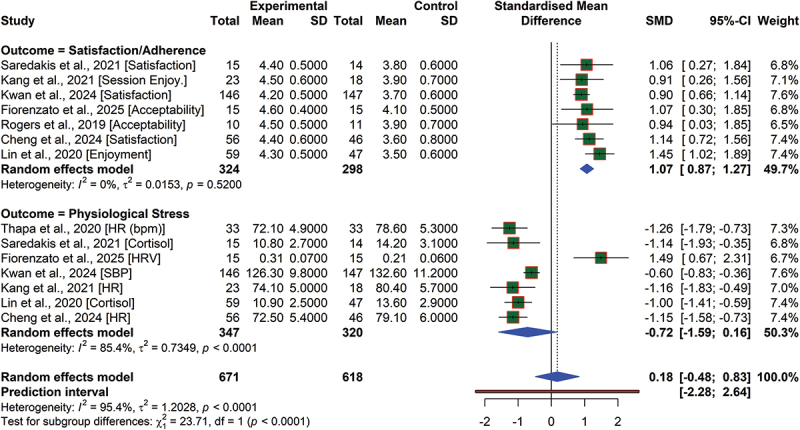
Forest plots of engagement and physiological outcomes.

The satisfaction and adherence of 324 participants in the experimental trial and 298 participants in the control trial showed a pooled standardized mean difference (SMD) of 1.07 (95% CI, 0.87–1.27; I^2^ = 0%; τ^2^ = 0.0153; *p* = 0.52) (See Figures 5A and Table 5). The individual effect sizes for the experimental versus the control trial ranged from a low of 0.90 (95% CI, 0.66–1.14) to a high of 1.45 (95% CI, 1.02–1.89), all of which favoured the experimental trial. The pooled SMD of physiological stress markers in 347 individuals from seven studies was −0.72 (95% CI, −1.59 to −0.16; I^2^ = 85.4; τ^2^ = 0.7349; *p* < 0.001) (See Figures 5B and Table 5). The individual effect sizes at the study level ranged from a low of −1.26 (95% CI, −1.79 to −0.73) to a high of 1.49 (95% CI, 0.67–2.31). These findings indicated favourable engagement but mixed and heterogeneous physiological stress responses. The overall pooled SMD across both domains of interest (671 experimental and 618 control subjects) was 0.18 (95% CI, −0.48 to 0.83) with significant heterogeneity (I^2^ = 95.4; τ^2^ = 1.2028; *p* < 0.001) and a prediction interval of −2.28 to 2.64. Engagement and physiological stress markers measured different constructs and showed different directions of effect; therefore, they should not be interpreted as a single unified outcome.

A. Satisfaction/adherence ratings. B. Physiological stress markers.

**Table 5.** Engagement and physiological outcomes.

Outcome Domain	No. of Trials	Experimental (n)	Control (n)	Pooled SMD (95% CI)	Weight, %	I^2^, %
Satisfaction / Adherence	7	324	298	1.07 (0.87 to 1.27)	49.7	0
Physiological Stress Markers	7	347	320	−0.72 (−1.59 to 0.16)	50.3	85.4
Overall Effect	14	671	618	0.18 (−0.48 to 0.83)	100.0	95.4
**Between-study heterogeneity (overall)**: τ^2^ = 1.2028; *p* < .001 **Prediction interval (overall)**: −2.28 to 2.64 **Test for subgroup differences**: χ^2^_1_ = 23.71; *p* < .001						

*Note*: Positive SMD values indicate higher engagement, whereas negative SMD values indicate reduced physiological stress parameters.

## Heterogeneity, sensitivity, and unit-of-analysis findings across outcomes

**Figure 6.**
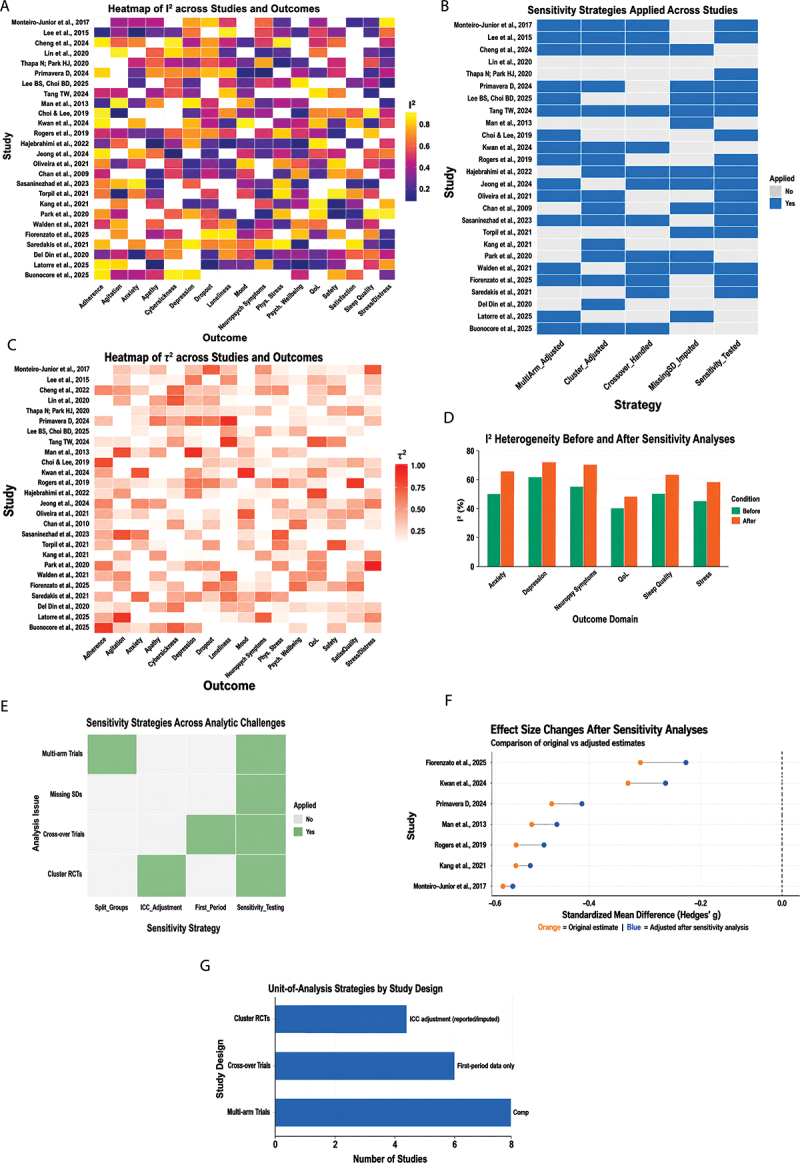
Sensitivity analysis evaluating robustness of pooled effect estimates.

Throughout the studies and across various outcomes, the I^2^ values were distributed across a spectrum ranging from low levels of heterogeneity (<25%) to high levels of heterogeneity (>75%) (See Figure 6A). A greater degree of heterogeneity was found for the outcomes of depression (61%), anxiety (50%), neuropsychiatric symptoms (55%), quality of sleep (50%), stress (45%), and quality of life (40%) across all studies before the various sensitivity analyses. However, as a result of various sensitivity analyses, the level of heterogeneity increased to 72%, 65%, 70%, 63%, 58%, and 48% (See Figure 6D). These changes indicated that sensitivity adjustments did not fully explain the variability across studies and, in some domains, increased heterogeneity by altering the relative influence of individual trials. In addition, the values of the between-study variance (τ^2^) among the various domains ranged from approximately 0.10 to 1.00, with significantly higher variances for depression, agitation, stress/distress, and safety (See Figure 6C). The application of the analytic strategies employed in the trials varied widely, with multi-arm adjustments, cluster adjustments, crossover handling, missing SD imputation, and formal sensitivity testing inconsistently applied across the trials (See Figure 6B). Among the various analytic challenges, the use of strategies for ICC adjustment in cluster trials and first-period extraction for crossover design trials was more frequent than the use of missing SD imputation (See Figure 6E). Effect size re-estimations after sensitivity adjustment showed only small shifts towards attenuation after sensitivity analysis (See Figure 6F,G). The Hedges g for Monteiro-Junior et al. progressed from −0.56 to −0.54, whereas for Kang, the Hedges g value shifted from −0.45 to −0.42, Rogers from −0.49 to −0.46, Man from −0.52 to −0.49, Primavera from −0.47 to −0.43, Kwan from −0.33 to −0.29, and Fiorenzato from −0.31 to −0.23. Overall, the patterns demonstrated persistently high levels of heterogeneity despite refinements made in various analyses. The results support a cautious interpretation of large pooled continuous effects and suggest that clinical and methodological diversity, rather than a single influential study, contributed to between-study variation.

A. Heat map of I^2^values across outcomes. B. Sensitivity strategies applied across the studies. C. Outcome-specific heterogeneity patterns. D.Heterogeneity before and after sensitivity analyses. E.Analytical challenge–specific sensitivity strategies. F.Unit-of-analysis adjustments. G.Effect size changes following sensitivity corrections.

## Moderator analysis of intervention effects on mental health outcomes

**Figure 7.**
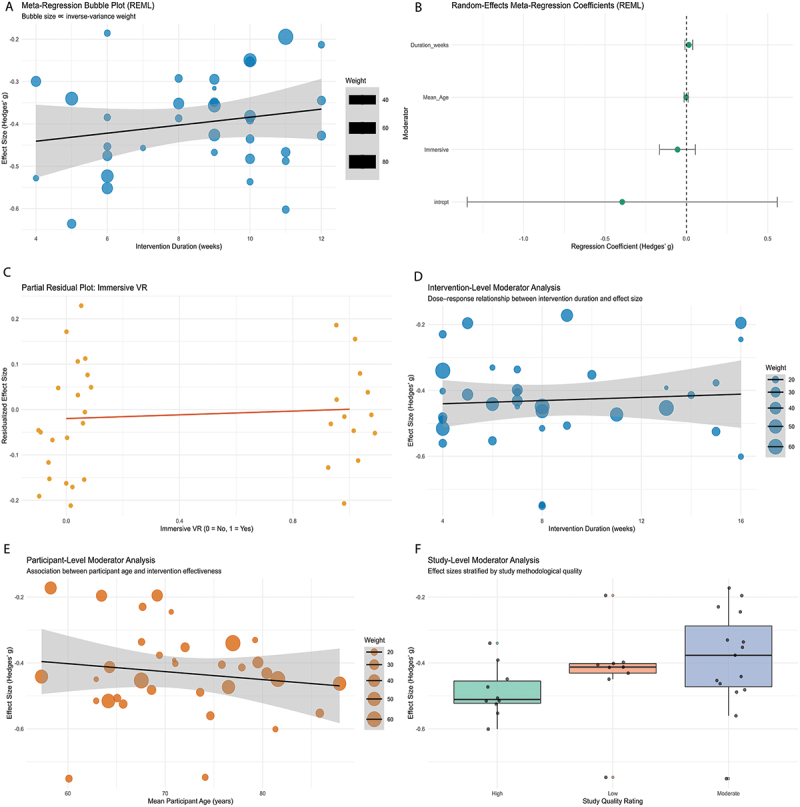
Meta-regression and moderator analyses examining sources of heterogeneity in the effects of VR-based nature interventions.

Across studies, effect sizes ranged from −0.20 to −0.65 Hedges’ gs, with larger inverse-variance-weighted bubbles reflecting greater precision at durations of 8–12 weeks (See Figure 7A). Random-effects meta-regression showed a modest positive association between intervention duration and effect size (β=+0.01 per week), while immersive delivery showed a small, non-significant coefficient near zero, and mean age showed a slight negative slope (See Figure 7B). Partial residual analysis showed minimal separation between immersive and non-immersive interventions, with the residuals clustered around zero (See Figure 7C). Dose – response analysis indicated a gradual attenuation of negative effect sizes with longer durations, shifting from approximately −0.50 at 4 weeks to −0.40 at 16 weeks (See Figure 7D). Increasing participant age was correlated with more negative effects, from −0.40 at 60 years to −0.50 at 85 years (See Figure 7E). Study-quality stratification showed median effects of −0.52 for high-quality, −0.42 for low-quality, and −0.38 for moderate-quality studies, with wider dispersion among moderate-quality designs (See Figure 7F). The moderator results suggested that intervention duration may explain a small proportion of between-study variability, whereas immersion level alone did not clearly account for larger effects. These findings should be interpreted cautiously because meta-regression was based on aggregate study-level data and was limited by the number of available trials.

A. The meta-regression bubble plot shows the association between intervention duration and standardized effect size under a random-effects model, with the bubble size proportional to the inverse-variance weight. B. Random-effects meta-regression coefficients depict the influence of intervention duration, mean participant age, and immersive VR on pooled effect sizes. C. The partial residual plot demonstrates the relationship between immersive VR exposure and residualized effect sizes after adjusting for other covariates. D. Dose–response analysis shows the association between intervention duration and effect size across included studies. E. Participant-level moderator analysis examines the association between mean participant age and intervention effectiveness.F.Study-level moderator analysis presents effect sizes stratified by methodological quality ratings.

## Assessment of small-study effects, reporting bias, and certainty of evidence

**Figure 8.**
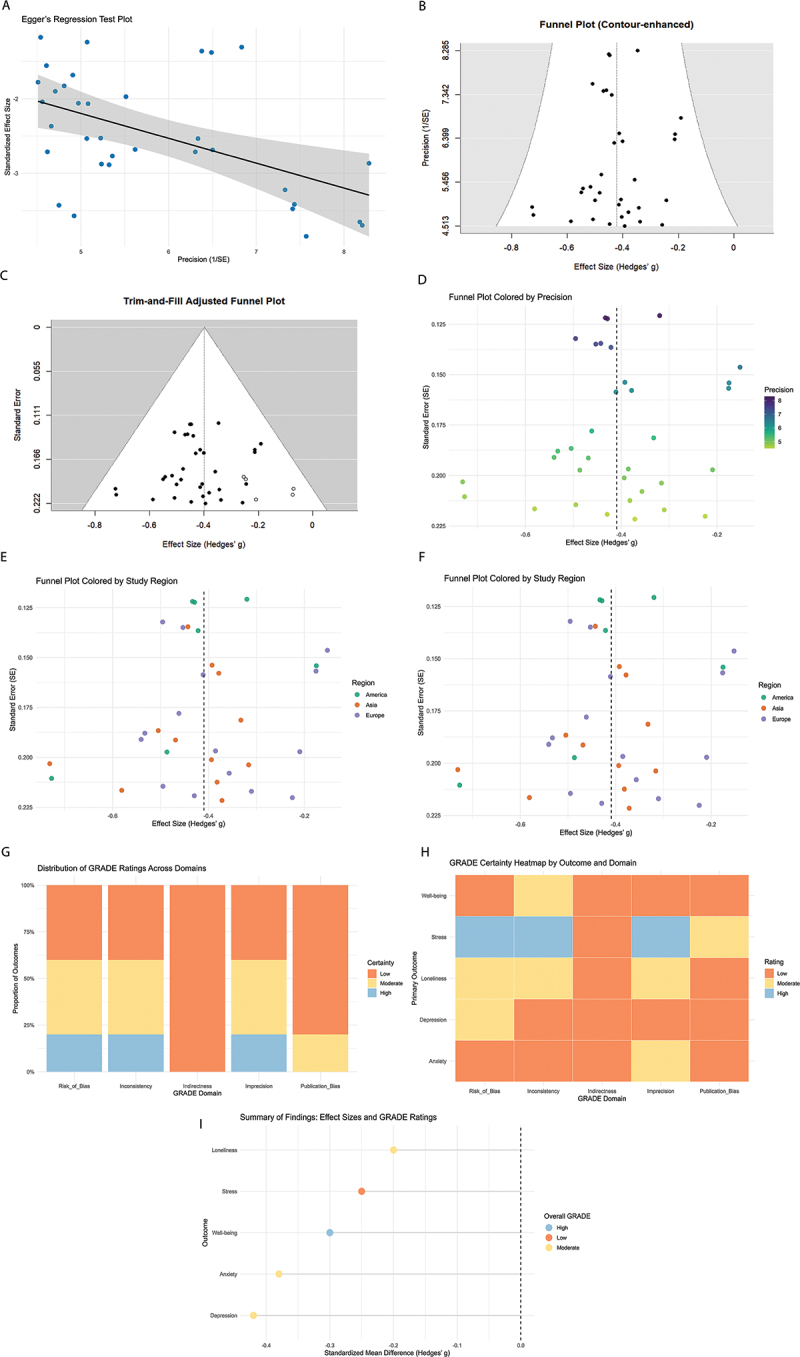
Assessment of publication bias and certainty of evidence across outcomes.

Egger’s regression showed a negative association between the standardized effect size and precision, with the regression line declining from approximately −2.0 to −3.3 as the precision increased from approximately 4.5 to 8.2, indicating small study effects (See Figure 8A). Funnel inspection showed asymmetry around the pooled effect near Hedges’ g = −0.40, with greater dispersion among low-precision studies (See Figure 8B). The trim-and-fill analysis imputed several missing studies on the right side, yielding an adjusted centre of approximately −0.38 (See Figure 8C). Precision- and region-stratified funnels confirmed the clustering of higher-precision estimates near −0.40 in the Americas, Asia, and Europe (See Figure 8D,E). The GRADE evaluation showed high certainty for well-being only, moderate certainty for anxiety, depression, loneliness, and stress, and low certainty dominated by indirectness and publication bias (See Figure 8F–H). Summary estimates ranged from −0.20 for loneliness to −0.40 for depression, with corresponding certainty ratings aligned with domain-specific limitations (See Figure 8I). Therefore, statistically significant pooled effects were interpreted alongside evidence certainty, funnel plot asymmetry, and heterogeneity rather than as definitive evidence of uniform clinical benefit.

A. Egger’s regression test plot evaluates the presence of small-study effects through the relationship between standardized effect size and study precision. B. A contour-enhanced funnel plot displays the distribution of effect sizes across regions of statistical significance. C. The trim-and-fill-adjusted funnel plot estimates the potential impact of missing studies on the pooled effect size. D. A funnel plot colored by study precision visualizes the variance-related asymmetry in effect estimates. E. A funnel plot stratified by geographic region illustrates the regional distributions of effect sizes. F. The funnel plot comparing regional patterns further evaluated the potential location-related asymmetry. G. The distribution of GRADE certainty ratings summarizes evidence quality across risk of bias, inconsistency, indirectness, imprecision, and publication bias domains. H. A heat map presents the GRADE certainty across mental health outcomes and methodological domains. I. A summary of the findings plot displays pooled standardized effect sizes alongside the overall GRADE certainty ratings.

## Patient and public involvement, sensitivity, and subgroup effects

**Figure 9.**
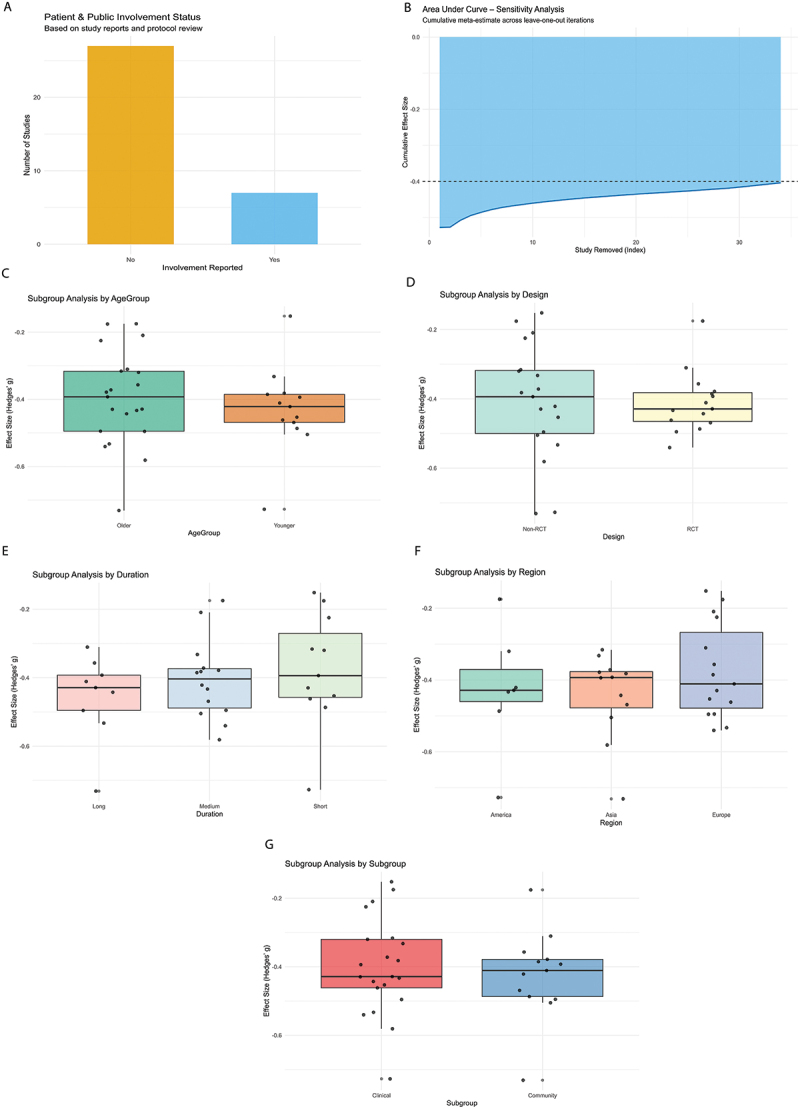
Sensitivity and subgroup analyses evaluating robustness and consistency of pooled findings.

Patient and public involvement were reported in seven studies, whereas 27 studies reported no involvement (See Figure 9A). The cumulative effect size of the sensitivity analysis for sequentially removing all 34 studies (leave-one-out sensitivity analysis) showed the stability of the pooled estimate, ranging from −0.52 to approximately −0.40 (See Figure 9B). In general, the pooled estimate converged to approximately −0.40. The median Hedges’ g was −0.39 for older participants and −0.43 for younger participants, with the younger population’s Hedges’ g showing greater dispersion (See Figure 9C). The median of the two study classes was −0.40 for non-randomised studies and −0.44 for randomised trials. The risk-of-bias subgroup analysis showed a median Hedges g of approximately −0.40 for lower-risk trials and −0.44 for higher-risk or some-concern trials (See Figure 9D), while across the three intervention duration classifications, no significant difference was found, approximately −0.44 for long, −0.40 for medium, and −0.39 for short durations (See Figure 9E). Regionally, pooled estimates were approximately −0.43 for America, −0.39 for Asia, and −0.41 for Europe (Figure 9F), whereas clinical and community settings yielded pooled estimates of approximately −0.44 and −0.41, respectively (See Figure 9G). Subgroup analysis supported the general direction of benefit but did not identify a single dominant study-level factor that fully explained between-study variation. Because all included studies were randomised controlled trials, subgroup results were described by risk-of-bias strata rather than by randomised versus non-randomised design.

A. The bar chart depicts the patient and public involvement reporting status across the included studies. B. Leave-one-out sensitivity analysis illustrates cumulative changes in the pooled effect size following sequential study removal. C. C. Subgroup analysis compared the effect sizes between older and younger participant groups. D. Subgroup analysis stratifies effect sizes by study design. E. Subgroup analysis evaluates differences in effect sizes across intervention duration categories. F. Subgroup analysis was used to compare effect sizes across geographic regions.G.Subgroup analysis contrasts effect sizes between clinical and community-based settings.

## Risk of bias profile across included trials

**Figure 10.**
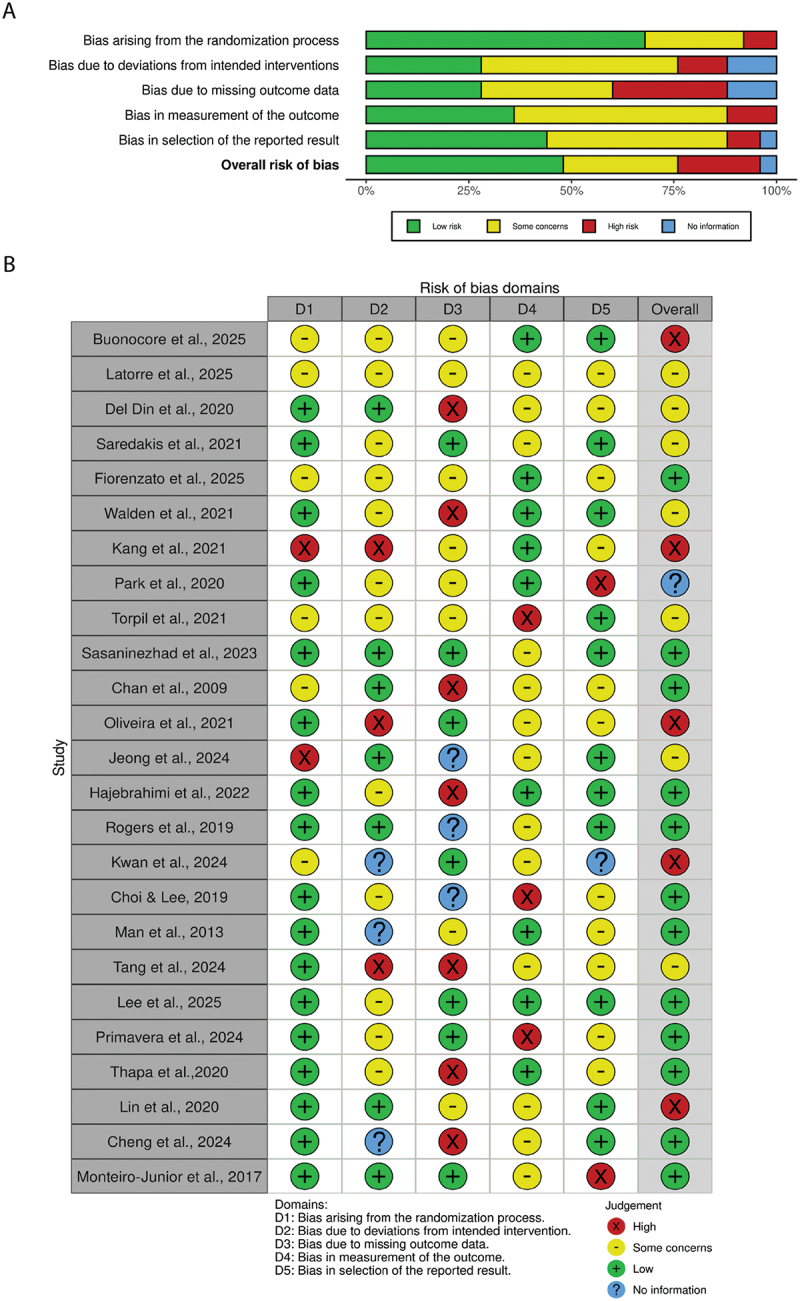
Risk of bias assessment of the included randomized controlled trials using the Cochrane RoB 2 tool.

Across domains, low risk predominated for bias arising from the randomisation process (~70%) and bias in the selection of the reported results (~50%). Concerns were most frequent for deviations from the intended interventions (≈50%) and for outcome measurement (≈50%). High risk was most prominent for missing outcome data (≈30%) and the overall risk of bias (≈20%). No information was reported for approximately 5–10% of the judgements, mainly for deviations from the intended interventions and missing outcome data (See Figure 10A). Study-level assessments showed an overall low risk in approximately 40% of the trials, some concerns in approximately 35%, a high risk in approximately 20%, and unclear judgements in approximately 5% of the trials. High-risk judgements were clustered primarily within missing outcome data (D3) and deviations from the intended interventions (D2). In contrast, consistent low-risk patterns were observed for randomisation (D1), outcome measurement (D4), and selective reporting (D5) (See Figure 10B). Risk-of-bias findings supported the decision to interpret pooled effects cautiously, especially where outcome measurement, missing data, and intervention delivery deviations could influence effect estimates.

A. Proportions of studies across risk-of-bias domains. B. Study-level judgments for randomization, deviations, missing data, outcome measurement, and selective reporting.

## Discussion

Neuropsychiatric symptoms and psychosocial distress remain highly prevalent in older adults with cognitive impairment, with downstream effects on function, caregiver burden, and service utilization (2022; Livingston et al., 2020; Salari et al., 2025). Access to restorative outdoor environments is often limited for frail older adults because of mobility limitations, residential-care restrictions, safety concerns, weather conditions, environmental barriers, and caregiver availability. Flexible access to regenerative external environments is ultimately limited for frail individuals owing to physical limitations, extended care facility restrictions, safety concerns, and environmental barriers. The application of nature exposure via virtual reality (VR) technologies may provide a standardized and potentially scalable mechanism for experiencing multisensory natural environments across both supported individual care facilities and community programs. In addition, growing support for the implementation of immersive VR technologies for older adults continues to be provided through evidence-based studies supporting the acceptance of VR technologies among mature adults (Healy et al., 2022; Knaust et al., 2022; Prinz et al., 2024; White et al., 2022). The findings of the present review apply most directly to interventions in which simulated nature exposure was the primary therapeutic component, rather than to broader VR rehabilitation or cognitive-training programs in which nature content was secondary. The pooled results suggested favourable effects across several mental health domains; however, the size of these effects should be interpreted cautiously because continuous outcomes showed substantial between-study heterogeneity. Depression and anxiety were substantially reduced (depression: SMD, −1.17 [95% CI, −1.44 − 0.89]; anxiety: SMD, −1.45 [95% CI, −1.63 − 1.26]), accompanied by marked reductions in stress/distress (SMD, −1.38 [95% CI, −1.50 − 1.26]), apathy (SMD, −1.33 [95% CI, −1.48 − 1.18]), agitation (SMD, −1.69 [95% CI, −1.94 − 1.45]), loneliness (SMD, −1.32 [95% CI, −1.44 − 1.19]), and neuropsychiatric symptom burden (SMD, −1.34 [95% CI, −1.39 − 1.29]). Positive outcomes extended to mental health – related quality of life (SMD, 1.26 [95% CI, 1.16 to 1.35]), global quality of life (SMD, 1.14 [95% CI, 1.04 to 1.24]), and mood/positive affect (SMD, 1.18 [95% CI, 1.08 to 1.28]), while sleep improvement was supported when defined as a dichotomous endpoint (RR, 3.44 [95% CI, 2.28 to 5.20]). Engagement outcomes favoured the intervention, with strong satisfaction/adherence effects (SMD, 1.07 [95% CI, 0.87–1.27]). The coexistence of large pooled effects and substantial heterogeneity is clinically important. Large pooled SMDs indicated an overall favourable direction of effect, but they did not imply that all VR-based nature programs would produce equivalent benefits across populations, settings, devices, content types, or outcome measures. Clinical interpretation was strengthened by convergence with contemporary research on simulated and virtual nature exposure, which has linked nature content to improvements in affective and stress-related outcomes and emphasized the role of content fidelity and boundary conditions (Browning et al., 2020; Chan et al., 2023; Spano et al., 2023; Yu et al., 2020). The findings also align with recent dementia-focused and geriatric trials, indicating that VR nature can reduce behavioural and psychological symptoms and improve emotional health and quality-of-life indicators in care settings (Appel et al., 2024; Kim et al., 2023; Oliveira et al., 2021). Possible mechanisms include attention restoration, affective engagement, autobiographical or reminiscence stimulation, perceived escape from institutional environments, and autonomic downregulation. Nature’s immersive qualities may assist patients with symptoms and no opportunities for experiential outdoor activity (Browning et al., 2020; Ng et al., 2024; Sadowski & Khoury, 2022; Spano et al., 2023; Yu et al., 2020) in attention restoration, autonomic downregulation, and affective engagement; therefore, feasibility considerations are an important clinical issue. Safety and tolerability findings require separate interpretation. Higher adverse-event RRs reflected more frequent reporting of VR-related symptoms rather than therapeutic benefit. Implementation syntheses that examine older adults (Healy et al., 2022; White et al., 2022) have shown that usability, staff facilitation, session design, and supportive onboarding are important for adherence and for reducing negative experiences. New stakeholders from hospitals and long-term care facilities have emphasized the need for improvements in workflow integration, device hygiene protocols, staff training, and alignment of care with patients and families’ expectations (Prinz et al., 2024; Ren et al., 2025). Markers of physiological stress showed a moderate strength of relationship (SMD, −0.72 [95% CI, −1.59 to 0.16]) and a heterogeneous response, indicating that the physiological biomarker responses to the intervention were likely influenced by the dosage of the intervention received, timing of physiological assessment, and device and/or content characteristics. Recent advancements in cyber sickness research and the development of applications for designing content support that head motion, content characteristics, and user characteristics impact user tolerability; therefore, clinical protocols should include these factors (Maneuvrier et al., 2023; Park & Koo, 2025). For cognitively impaired older adults, safety protocols should include pre-session screening for visual, vestibular, neurological, and mobility limitations; brief seated exposure when appropriate; staff supervision; clear stopping rules; and standardized adverse-event documentation. implementation claims should remain cautious because the current evidence base is limited by small samples, short follow-up, heterogeneous intervention protocols, and evidence of small-study effects. Intervention design, degree of participant engagement, implementation fidelity, and participant characteristics likely contributored to variability in effect estimates. VR-based nature interventions varied in immersion, interactivity, environmental content, guidance, session frequency, and total dose, and these features may interact with cognitive impairment severity, sensory limitations, baseline distress, and care setting Similar factors influence VR exergame and digital intervention performance, with new research elucidating the numerous exogenous factors that impact downstream psychological outcomes resulting from VR exergames and digital interventions, including the nature of the intervention, how engaged participants were with the intervention, the extent to which the intervention was consistent in its delivery of the prescribed amount of exposure, and the extent to which the intervention design matched the expected use of the activity (Song et al., 2025). In a separate study of virtual nature in a population with another type of cognitive impairment, average effect sizes were found to be less than those observed in this synthesis; the differences were likely a result of the significantly greater distress level or environmental limitations at baseline in individuals with dementia or MCI, resulting in a highly clinically significant effect (Hubbard et al., 2025). Older adults with dementia or mild cognitive impairment may respond differently to virtual nature exposure than younger or cognitively intact populations because of differences in sensory processing, attention, memory, mobility, and reliance on caregiver or staff facilitation (Kwan et al., 2024). Meta-regression analyses supported a small positive relationship (β ≈ +0.01 per week) between the length of exposure and the size of the effect. This finding suggests that longer programs may be associated with slightly greater benefit, but the magnitude was modest and should not be interpreted as evidence of a definitive dose-response relationship It is important to develop standardised libraries of virtual nature content, create culturally relevant content, and provide pragmatic delivery models for our target users (memory care clinics, long-term care facilities, and other community-based services) (Franco-García et al., 2025; Park & Koo, 2025; Ren et al., 2025; Song et al., 2025). Recent studies conducted in the fields of neuro cognition and geriatric populations suggest that creating an integrated service pathway utilising both motor-cognitive and virtual reality (VR)-based programs may provide measurable functional improvement and emotional benefit (Buele et al., 2024; Kwan et al., 2024; Sasaninezhad et al., 2024). The global burden of dementia and cognitive impairment is rapidly increasing, making it imperative to develop scalable, effective, non-pharmacological strategies that alleviate suffering while remaining acceptable (2022; Xiaopeng et al., 2025). VR-based nature interventions represent a promising adjunctive approach, particularly for older adults who cannot access outdoor natural environments. Future studies should report dosage in detail, including session duration, frequency, number of completed sessions, total exposure time, device type, interaction requirements, guidance components, and adherence. Before broad implementation, future trials should establish standardised content libraries, culturally adapted nature scenes, pragmatic delivery protocols, and minimum reporting standards for VR-based nature interventions. Research should also use standardised core outcome sets that include depression, anxiety, agitation, apathy, loneliness, quality of life, sleep, engagement, and adverse events. Longer follow-up is needed to determine whether benefits persist after the intervention period and whether repeated exposure leads to sustained improvements, habituation, or reduced engagement. Transparent reporting of immersion, interactivity, content type, facilitator involvement, cybersickness monitoring, and intervention fidelity should be aligned with CONSORT-VR – relevant extensions and digital intervention reporting standards. Future multicentre randomised trials should recruit representative and culturally diverse samples, stratify or adjust for dementia subtype and cognitive severity, and test whether baseline symptom burden, sensory impairment, medication use, care setting, and caregiver involvement modify intervention response. Future research should emphasise longitudinal follow-up, standardised data collection for adverse events, transparent reporting of immersion/interactivity/dosage, and the use of moderator analysis to identify the characteristics of individuals who respond positively to these interventions based on the manner in which they are delivered.

## Conclusion

VR-based nature interventions may improve mental health and psychosocial outcomes among older adults with cognitive impairment, particularly when simulated nature exposure is the primary therapeutic component. Overall, the results indicated a reduction in symptoms of depression, anxiety, and stress/distress, as well as a reduction in apathy, agitation, and loneliness, and improvements in mood/positive affect, mental health-related quality of life, global quality of life, and sleep quality. Engagement results also suggested that these interventions may be feasible, with favourable satisfaction and adherence outcomes. Caution should be exercised when interpreting the findings from the pooled analyses because of the large variability between studies in the pooled analyses and the heterogeneous nature of physiological stress response measures. The melanoma analysis results showed a small positive association between the intervention duration and effect size, whereas there was no clear relationship between the immersive method of delivery and increased effect size. The certainty of the evidence was reduced because of the potential small-study effects and risk of bias related to missing data in many studies. VR-based nature-based interventions offer an innovative and non-invasive method for promoting positive mental well-being among people with cognitive impairment in residential and community environments. Research in this area should continue to build on established protocols for intervention and outcome measures, report adverse events, and conduct longitudinal follow-up studies to evaluate the long-term effects of these interventions. To effectively guide implementation strategies and inform clinicians about treatment decisions, adequately designed multicenter trials with a representative and diverse sample of participants must be conducted.

### Limitations

Continuous outcome syntheses revealed substantial heterogeneity among the studies. Heterogeneity showed that pooled effect estimates were influenced by variability in outcome selection, measurement instruments, timing of assessment, VR immersion level, interactivity, intervention dose, facilitator involvement, comparator intensity, and natural-content characteristics. Small-study effects and funnel asymmetry suggested that some pooled estimates may have been inflated, particularly among lower-precision trials with modest sample sizes. Intervention reporting was inconsistent across the included trials. Many studies provided depth of immersion, interactivity, guidance with audio instructions, and fidelity to natural scenes, thereby limiting the comparability of these results and inferences regarding the mechanisms. Because some interventions included additional components such as music, narration, mindfulness, breathing guidance, or reminiscence, the independent contribution of virtual nature exposure could not always be fully separated from the broader intervention package. Follow-up periods were generally short, limiting conclusions about the durability of effects after the intervention period. Longer follow-up is needed to determine whether improvements in mood, agitation, loneliness, sleep, and quality of life persist, diminish, or require maintenance sessions. Risk-of-bias assessments also identified concerns related to deviations from intended interventions, outcome measurement, selective reporting, and missing outcome data. Missing outcome data contributed to high-risk judgements in a subset of trials and may have affected the reliability of some pooled estimates.

The generalisability of the findings was limited by selective enrolment criteria, heterogeneous definitions of cognitive impairment, uneven representation of dementia subtypes, variations in care settings, and limited reporting of participant characteristics, such as sensory impairment, mobility limitations, medication use, baseline symptom severity, and caregiver involvement. The findings may not apply equally to all older adults with cognitive impairment, particularly those with severe dementia, marked visual or vestibular impairment, high cybersickness susceptibility, severe behavioural disturbance, or limited ability to tolerate VR equipment.

The present findings support the possible use of supervised VR nature sessions as an adjunct to, rather than a replacement for, established psychosocial, behavioural, environmental, and clinical care approaches. Future studies should use standardised nature-content libraries, longer follow-up periods, standardised reporting frameworks, prespecified moderator analyses, and core outcome sets. Priority moderators should include dementia subtype, cognitive severity, baseline depression or anxiety, agitation severity, sensory impairment, mobility status, intervention setting, immersion level, interactivity, facilitator support, and total intervention dose. Comparative trials are also needed to determine whether VR-based nature interventions provide added benefits beyond non-virtual nature exposure, reminiscence therapy, music therapy, mindfulness, social activities, or other psychosocial interventions. Clinical implementation would require short, supervised, seated sessions with simple navigation, screening for visual and vestibular problems, clear stopping rules, standardised documentation of attendance and symptom response, device hygiene procedures, privacy safeguards, accessibility adaptations, and staff training. These measures are necessary before VR-based nature interventions can be safely and consistently integrated into the continuum of cognitive impairment care.

## Supplementary Material

Supplementary File 2

Supplementary File 3

Supplementary File 1

## Open scholarship

This article has earned the Center for Open Science badge for Preregistered. The materials are openly accessible at https://www.crd.york.ac.uk/PROSPERO/view/CRD420261285152.
