# Supplementary material for: Effectiveness of virtual reality-based nature interventions in promoting mental health outcomes among older adults with cognitive impairment: a systematic review and meta-regression analysis
Source: Aust J Psychol. 2026 Jun 15;78(1):2679707. doi: 10.1080/00049530.2026.2679707 (PMC13270873; doi:10.1080/00049530.2026.2679707)
Supplement: Supplementary File 2 [file RAUP_A_2679707_SM9607.docx]

**Table. 1. Database-specific search strategy using MeSH terms, keywords, and Boolean operators**

| **Database** | **Controlled vocabulary (subject headings)** | **Free-text keywords (Title/Abstract/Keywords)** | **Boolean search string (copy/paste-ready)** |
| --- | --- | --- | --- |
| **MEDLINE via PubMed** | “Virtual Reality”[MeSH]; “Aged”[MeSH]; “Cognitive Dysfunction”[MeSH]; “Mild Cognitive Impairment”[MeSH]; “Dementia”[MeSH]; “Depression”[MeSH]; “Anxiety”[MeSH]; “Stress, Psychological”[MeSH]; “Agitation”[MeSH]; “Apathy”[MeSH]; “Loneliness”[MeSH]; “Quality of Life”[MeSH]; “Sleep”[MeSH] | virtual reality OR VR OR immersive OR head-mounted display OR HMD OR 360 video; nature OR natural environment* OR green space* OR greenspace* OR blue space* OR forest* OR park* OR garden* OR “natural scene*”; older adult* OR aged OR elderly OR geriatric*; cognitive impairment OR cognitive dysfunction OR mild cognitive impairment OR MCI OR dementia OR Alzheimer*; depression OR depressive OR anxiety OR stress OR distress OR agitation OR apathy OR mood OR affect OR loneliness OR wellbeing OR “quality of life” OR sleep | ((“Virtual Reality”[MeSH] OR “virtual reality”[tiab] OR VR[tiab] OR immersive[tiab] OR “head-mounted display”[tiab] OR HMD[tiab] OR “360 video”[tiab] OR “360-degree”[tiab]) AND (nature[tiab] OR “natural environment*”[tiab] OR “green space*”[tiab] OR greenspace*[tiab] OR “blue space*”[tiab] OR forest*[tiab] OR park*[tiab] OR garden*[tiab] OR “natural scene*”[tiab]) AND (“Aged”[MeSH] OR older adult*[tiab] OR aged[tiab] OR elderly[tiab] OR geriatric*[tiab]) AND (“Cognitive Dysfunction”[MeSH] OR “Mild Cognitive Impairment”[MeSH] OR “Dementia”[MeSH] OR cognitive impairment[tiab] OR cognitive dysfunction[tiab] OR MCI[tiab] OR dementia[tiab] OR Alzheimer*[tiab]) AND (“Depression”[MeSH] OR “Anxiety”[MeSH] OR “Stress, Psychological”[MeSH] OR “Agitation”[MeSH] OR “Apathy”[MeSH] OR “Loneliness”[MeSH] OR “Quality of Life”[MeSH] OR “Sleep”[MeSH] OR depression[tiab] OR anxiety[tiab] OR stress[tiab] OR distress[tiab] OR agitation[tiab] OR apathy[tiab] OR mood[tiab] OR affect[tiab] OR loneliness[tiab] OR wellbeing[tiab] OR “quality of life”[tiab] OR sleep[tiab])) AND (randomized controlled trial[pt] OR controlled clinical trial[pt] OR random*[tiab] OR trial[tiab]) AND (English[lang]) AND (“2020/01/01”[dp] : “2025/12/31”[dp]) |
| **Embase via Ovid** | virtual reality/; aged/; cognitive defect/ OR cognitive impairment/; mild cognitive impairment/; dementia/; depression/; anxiety/; psychological stress/; agitation/; apathy/; loneliness/; quality of life/; sleep/ | virtual reality OR VR OR immersive OR head-mounted display OR HMD OR 360 video; nature OR natural environment* OR green space* OR greenspace* OR blue space* OR forest* OR park* OR garden* OR natural scene*; older adult* OR elderly OR geriatric*; cognitive impairment OR cognitive dysfunction OR mild cognitive impairment OR MCI OR dementia OR Alzheimer*; depression OR anxiety OR stress OR distress OR agitation OR apathy OR mood OR affect OR loneliness OR wellbeing OR quality of life OR sleep | (('virtual reality'/exp OR 'virtual reality':ti,ab,kw OR VR:ti,ab,kw OR immersive:ti,ab,kw OR 'head mounted display':ti,ab,kw OR HMD:ti,ab,kw OR '360 video':ti,ab,kw OR '360-degree':ti,ab,kw) AND (nature:ti,ab,kw OR 'natural environment*':ti,ab,kw OR 'green space*':ti,ab,kw OR greenspace*:ti,ab,kw OR 'blue space*':ti,ab,kw OR forest*:ti,ab,kw OR park*:ti,ab,kw OR garden*:ti,ab,kw OR 'natural scene*':ti,ab,kw) AND ('aged'/exp OR older NEXT/1 adult*:ti,ab,kw OR elderly:ti,ab,kw OR geriatric*:ti,ab,kw) AND (('mild cognitive impairment'/exp OR 'dementia'/exp OR 'cognitive defect'/exp) OR 'cognitive impairment':ti,ab,kw OR 'cognitive dysfunction':ti,ab,kw OR MCI:ti,ab,kw OR dementia:ti,ab,kw OR Alzheimer*:ti,ab,kw) AND (depression/exp OR anxiety/exp OR 'psychological stress'/exp OR agitation/exp OR apathy/exp OR loneliness/exp OR 'quality of life'/exp OR sleep/exp OR depression:ti,ab,kw OR anxiety:ti,ab,kw OR stress:ti,ab,kw OR distress:ti,ab,kw OR agitation:ti,ab,kw OR apathy:ti,ab,kw OR mood:ti,ab,kw OR affect:ti,ab,kw OR loneliness:ti,ab,kw OR wellbeing:ti,ab,kw OR 'quality of life':ti,ab,kw OR sleep:ti,ab,kw)) AND (random*:ti,ab,kw OR trial*:ti,ab,kw OR 'randomized controlled trial'/exp) AND [english]/lim AND [2020-2025]/py |
| **PsycINFO** | DE “Virtual Reality”; DE “Aged”; DE “Cognitive Impairment”; DE “Mild Cognitive Impairment”; DE “Dementia”; DE “Depression”; DE “Anxiety”; DE “Stress”; DE “Agitation”; DE “Apathy”; DE “Loneliness”; DE “Quality of Life”; DE “Sleep” | virtual reality OR VR OR immersive OR head-mounted display OR HMD OR 360 video; nature OR natural environment* OR green space* OR blue space* OR forest* OR park* OR garden*; older adult* OR elderly OR geriatric*; cognitive impairment OR MCI OR dementia OR Alzheimer*; depression OR anxiety OR stress OR agitation OR apathy OR mood OR loneliness OR wellbeing OR quality of life OR sleep | ((DE “Virtual Reality” OR TI,AB(“virtual reality” OR VR OR immersive OR “head-mounted display” OR HMD OR “360 video” OR “360-degree”)) AND (TI,AB(nature OR “natural environment*” OR “green space*” OR greenspace* OR “blue space*” OR forest* OR park* OR garden* OR “natural scene*”)) AND (DE “Aged” OR TI,AB(older adult* OR aged OR elderly OR geriatric*)) AND (DE “Mild Cognitive Impairment” OR DE “Dementia” OR DE “Cognitive Impairment” OR TI,AB(“cognitive impairment” OR “cognitive dysfunction” OR MCI OR dementia OR Alzheimer*)) AND (DE “Depression” OR DE “Anxiety” OR DE “Stress” OR DE “Agitation” OR DE “Apathy” OR DE “Loneliness” OR DE “Quality of Life” OR DE “Sleep” OR TI,AB(depression OR anxiety OR stress OR distress OR agitation OR apathy OR mood OR affect OR loneliness OR wellbeing OR “quality of life” OR sleep))) AND (TI,AB(random* OR trial) OR DE “Treatment Effectiveness Evaluation” OR DE “Clinical Trials”) AND English AND PY 2020-2025 |
| **CINAHL** | (MH “Virtual Reality+”); (MH “Aged+”); (MH “Cognitive Impairment” OR MH “Mild Cognitive Impairment” OR MH “Dementia+”); (MH “Depression+” OR MH “Anxiety+” OR MH “Stress, Psychological” OR MH “Agitation” OR MH “Apathy” OR MH “Loneliness” OR MH “Quality of Life+” OR MH “Sleep”) | virtual reality OR VR OR immersive OR head-mounted display OR HMD OR 360 video; nature OR natural environment* OR green space* OR blue space* OR forest* OR park* OR garden*; older adult* OR elderly OR geriatric*; cognitive impairment OR MCI OR dementia OR Alzheimer*; depression OR anxiety OR stress OR distress OR agitation OR apathy OR mood OR loneliness OR wellbeing OR quality of life OR sleep | ((MH “Virtual Reality+” OR TI (“virtual reality” OR VR OR immersive OR “head-mounted display” OR HMD OR “360 video” OR “360-degree”) OR AB (“virtual reality” OR VR OR immersive OR “head-mounted display” OR HMD OR “360 video” OR “360-degree”)) AND (TI (nature OR “natural environment*” OR “green space*” OR greenspace* OR “blue space*” OR forest* OR park* OR garden* OR “natural scene*”) OR AB (nature OR “natural environment*” OR “green space*” OR greenspace* OR “blue space*” OR forest* OR park* OR garden* OR “natural scene*”)) AND (MH “Aged+” OR TI (older adult* OR aged OR elderly OR geriatric*) OR AB (older adult* OR aged OR elderly OR geriatric*)) AND ((MH “Cognitive Impairment” OR MH “Mild Cognitive Impairment” OR MH “Dementia+”) OR TI (“cognitive impairment” OR “cognitive dysfunction” OR MCI OR dementia OR Alzheimer*) OR AB (“cognitive impairment” OR “cognitive dysfunction” OR MCI OR dementia OR Alzheimer*)) AND ((MH “Depression+” OR MH “Anxiety+” OR MH “Stress, Psychological” OR MH “Agitation” OR MH “Apathy” OR MH “Loneliness” OR MH “Quality of Life+” OR MH “Sleep”) OR TI (depression OR anxiety OR stress OR distress OR agitation OR apathy OR mood OR affect OR loneliness OR wellbeing OR “quality of life” OR sleep) OR AB (depression OR anxiety OR stress OR distress OR agitation OR apathy OR mood OR affect OR loneliness OR wellbeing OR “quality of life” OR sleep))) AND (PT “Randomized Controlled Trial” OR TI random* OR AB random*) AND English AND (YR 2020-2025) |
| **Scopus** | No MeSH (keyword-based indexing) | virtual reality OR VR OR immersive OR head-mounted display OR HMD OR 360 video; nature OR natural environment* OR green space* OR blue space* OR forest* OR park* OR garden* OR “natural scene*”; older adult* OR aged OR elderly OR geriatric*; cognitive impairment OR MCI OR dementia OR Alzheimer*; depression OR anxiety OR stress OR agitation OR apathy OR mood OR loneliness OR wellbeing OR “quality of life” OR sleep; randomized OR trial | (TITLE-ABS-KEY(("virtual reality" OR VR OR immersive OR "head-mounted display" OR HMD OR "360 video" OR "360-degree") AND (nature OR "natural environment*" OR "green space*" OR greenspace* OR "blue space*" OR forest* OR park* OR garden* OR "natural scene*") AND ("older adult*" OR aged OR elderly OR geriatric*) AND ("cognitive impairment" OR "cognitive dysfunction" OR "mild cognitive impairment" OR MCI OR dementia OR Alzheimer*) AND (depression OR anxiety OR stress OR distress OR agitation OR apathy OR mood OR affect OR loneliness OR wellbeing OR "quality of life" OR sleep) AND (random* OR trial))) AND (LIMIT-TO(LANGUAGE, "English")) AND (PUBYEAR > 2019 AND PUBYEAR < 2026) |
| **Web of Science Core Collection** | No MeSH (topic-based indexing) | virtual reality OR VR OR immersive OR head-mounted display OR HMD OR 360 video; nature OR natural environment* OR green space* OR blue space* OR forest* OR park* OR garden*; older adult* OR aged OR elderly OR geriatric*; cognitive impairment OR MCI OR dementia OR Alzheimer*; depression OR anxiety OR stress OR agitation OR apathy OR mood OR loneliness OR wellbeing OR quality of life OR sleep; randomized OR trial | TS=(("virtual reality" OR VR OR immersive OR "head-mounted display" OR HMD OR "360 video" OR "360-degree") AND (nature OR "natural environment*" OR "green space*" OR greenspace* OR "blue space*" OR forest* OR park* OR garden* OR "natural scene*") AND ("older adult*" OR aged OR elderly OR geriatric*) AND ("cognitive impairment" OR "cognitive dysfunction" OR "mild cognitive impairment" OR MCI OR dementia OR Alzheimer*) AND (depression OR anxiety OR stress OR distress OR agitation OR apathy OR mood OR affect OR loneliness OR wellbeing OR "quality of life" OR sleep) AND (random* OR trial)) Refined by: LANGUAGES=(ENGLISH) AND PUBLICATION YEARS=(2020-2025) |
| **Cochrane CENTRAL** | Cochrane indexing (no MeSH required, but MeSH-compatible terms work) | virtual reality OR VR OR immersive OR head-mounted display OR 360 video; nature OR natural environment* OR green space* OR blue space* OR forest* OR park* OR garden*; older adult* OR aged OR elderly; cognitive impairment OR MCI OR dementia OR Alzheimer*; depression OR anxiety OR stress OR agitation OR apathy OR mood OR loneliness OR wellbeing OR quality of life OR sleep | ("virtual reality" OR VR OR immersive OR "head-mounted display" OR HMD OR "360 video" OR "360-degree") AND (nature OR "natural environment*" OR "green space*" OR greenspace* OR "blue space*" OR forest* OR park* OR garden* OR "natural scene*") AND ("older adult*" OR aged OR elderly OR geriatric*) AND ("cognitive impairment" OR "cognitive dysfunction" OR "mild cognitive impairment" OR MCI OR dementia OR Alzheimer*) AND (depression OR anxiety OR stress OR distress OR agitation OR apathy OR mood OR affect OR loneliness OR wellbeing OR "quality of life" OR sleep) |
| **ClinicalTrials.gov** | Registry fields (no MeSH) | virtual reality OR VR; nature OR natural environment OR forest OR park OR garden; older adult OR elderly; mild cognitive impairment OR dementia OR Alzheimer; depression OR anxiety OR agitation OR wellbeing | (("virtual reality" OR VR) AND (nature OR "natural environment" OR forest OR park OR garden) AND (older OR elderly OR "older adult") AND ("mild cognitive impairment" OR MCI OR dementia OR Alzheimer)) |
| **WHO ICTRP** | Registry fields (no MeSH) | virtual reality OR VR; nature OR natural environment OR green space OR forest OR park OR garden; older adult OR elderly; mild cognitive impairment OR dementia OR Alzheimer | ("virtual reality" OR VR) AND (nature OR "natural environment" OR "green space" OR forest OR park OR garden) AND (older OR elderly OR "older adult") AND ("mild cognitive impairment" OR MCI OR dementia OR Alzheimer) |
| **Google Scholar** | No MeSH | “virtual reality” OR VR OR immersive; nature OR “natural environment” OR greenspace OR “green space” OR “blue space” OR forest OR park OR garden; “older adults” OR elderly; “cognitive impairment” OR MCI OR dementia OR Alzheimer; depression OR anxiety OR agitation OR loneliness OR wellbeing OR “quality of life” OR sleep; randomized OR trial | ("virtual reality" OR VR OR immersive OR "head-mounted display" OR HMD OR "360 video") AND (nature OR "natural environment" OR greenspace OR "green space" OR "blue space" OR forest OR park OR garden OR "natural scene") AND ("older adults" OR elderly OR aged OR geriatric) AND ("cognitive impairment" OR "mild cognitive impairment" OR MCI OR dementia OR Alzheimer) AND (depression OR anxiety OR stress OR agitation OR apathy OR mood OR loneliness OR wellbeing OR "quality of life" OR sleep) AND (randomized OR randomised OR trial) |
